# Multi-omics characterization of IDH-mutant astrocytoma-derived cell lines reveals NOTCH-regulated plastic quiescent astrocyte-like state and insights into progression

**DOI:** 10.1186/s40478-026-02316-9

**Published:** 2026-07-20

**Authors:** Leonor Garcia, Christine Granotier-Beckers, Donovan Pineau, Dassou Sika, Szimonetta Hideg, Camille Labadie, Kasandra Aguilar Cázarez, Jigna Kundnani, Lucille Stuani, Laurent R. Gauthier, François D. Boussin, Marc-André Mouthon, Serge Urbach, Khadija El Koulali, Martial Séveno, Chantal Ripoll, Kawtar Daddi, Maité Verreault, Ahmed Idbaih, Samantha O’Connor, Christopher L. Plaisier, Wei Chen, Sheng-Qing Lv, Lei Zhang, Min Zheng, Valérie Rigau, Hugues Duffau, Jean-Philippe Hugnot

**Affiliations:** 1 IGF, Univ Montpellier, CNRS, Inserm, Montpellier, France; 2 Inserm, CEA, Stabilité Génétique Cellules Souches Et Radiations, LRP/iRCM/IBFJ, Université Paris Cité, 92265 Fontenay-Aux-Roses, France; 3 Inserm, CEA, Stabilité Génétique Cellules Souches Et Radiations, LRP/iRCM/IBFJ, Université Paris-Saclay, 92265 Fontenay-Aux-Roses, France; 4 Institut de Génétique Humaine (IGH), CNRS, Université de Montpellier, Montpellier, France; 5 Université de Montpellier, Montpellier, France; 6 Institut de Recherche en Cancérologie (IRCM), INSERM, Montpellier, France; 7 Equipe Labélisée Ligue Contre Le Cancer, Paris, France; 8 BCM, CNRS, INSERM, Université Montpellier, Montpellier, France; 9 AP-HP, Institut du Cerveau (ICM), CNRS, INSERM, Hôpitaux Universitaires La Pitié Salpêtrière - Charles Foix, Service de Neuro-Oncologie-Institut de Neurologie, Sorbonne Université, 75013 Paris, France; 10 School of Biological and Health Systems Engineering, Arizona State University, Tempe, AZ USA; 11 Jinfeng Laboratory, Chongqing, China; 12 Department of Neurosurgery, Xinqiao Hospital, Third Military Medical University (Army Medical University), Chongqing, China; 13 CHU-Gui de Chauliac, Montpellier, France

**Keywords:** Low-grade glioma, Astrocytoma, IDH1 mutation, Cell line biobank, Cellular heterogeneity, Cell plasticity, Quiescence, NOTCH pathway

## Abstract

Diffuse IDH-mutant astrocytomas are brain tumors typically diagnosed as low-grade but capable of progressing to higher grades. They exhibit three cellular states resembling astrocytes, oligodendrocytes, and neural progenitor (NPC) cells. Understanding their biology has been limited by the scarcity of relevant in vitro models. Here, we established and extensively characterized four astrocytoma cell lines (LGG275, LGG336, LGG85, LGG349) derived from IDH-mutant astrocytomas of different grades and analyzed them using multi-omics approaches. These lines display growth rates in vitro and in vivo consistent with tumor grade and recapitulate some key molecular alterations observed in patient tumors, including *IDH1*, *ATRX*, and *TP53* mutations, ALT (alternative lengthening of telomeres) activation and, in more aggressive lines, MET and PDGFRA alterations. Single-cell RNA sequencing revealed three major transcriptional states (astrocyte-like, oligodendrocyte-like, and NPC-like), consistent with those described in patient tumors. A hallmark of higher-grade-derived lines (LGG85, LGG349) is the persistence of NPC-like populations without growth factors possibly reflecting tumor progression. The LGG275 line most accurately mirrors slow-growing astrocytomas. Using CD44 and GLAST, we isolated astrocyte-like (CD44⁺/GLAST⁺) cells from LGG275 that preferentially adopt a quiescent state yet retain remarkable plasticity, generating oligodendrocyte-like cells (CD44⁻/GLAST⁻). Transcriptomic and proteomic analyses revealed that astrocyte-like and oligodendrocyte-like cells populations resemble, respectively, quiescent and activated neural stem (NSC) cells from the adult subventricular zone (SVZ). Finally, we provide evidence that NOTCH signaling contributes to the regulation of cell state balance, promoting transitions toward an astrocyte-like transcriptional program while DLL3, an anti-Notch protein, expressed by oligodendrocyte-like cells, modulates both proliferation and phenotype. These cell lines represent valuable resources for dissecting lineage dynamics, heterogeneity, and progression mechanisms in IDH-mutant astrocytomas.

**Graphical abstract:**

**Figure:**
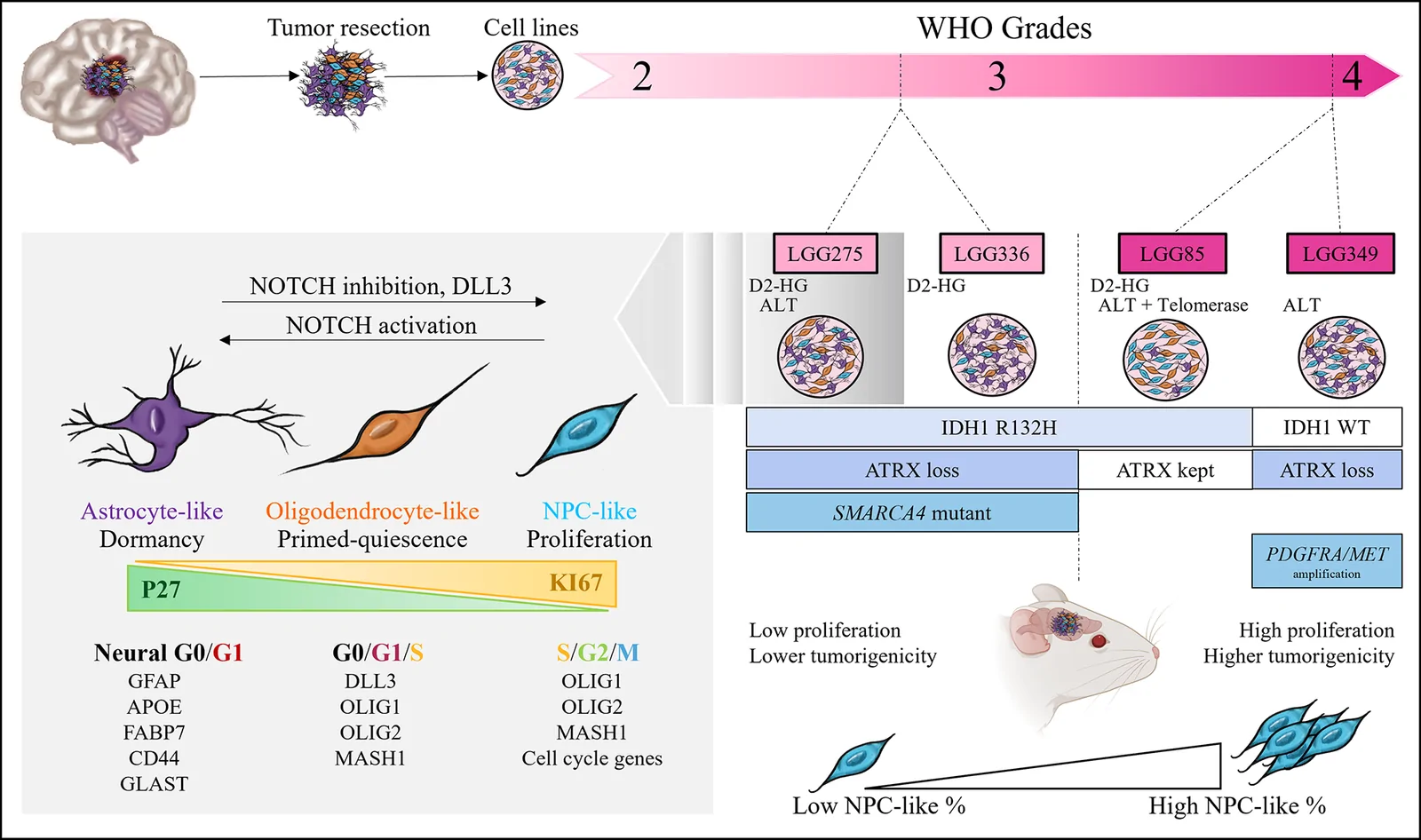

**Supplementary Information:**

The online version contains supplementary material available at https://doi.org/10.1186/s40478-026-02316-9.

## Introduction

Diffuse low-grade gliomas (DLGG) are rare primary brain tumors that mainly affect young adults [1]. Approximately 80% harbor IDH1 or IDH2 mutations, leading to production of the oncometabolite 2-hydroxyglutarate (2-HG), which disrupts epigenetic regulation and impairs differentiation [2, 3]. According to the WHO classification IDH-mutant gliomas include astrocytomas (characterized by *ATRX* and *TP53* alterations and frequently associated with ALT) and oligodendrogliomas defined by 1p/19q co-deletion and *CIC/FUBP1* mutations [4]. Although typically slow-growing and diagnosed at grade 2, these tumors can progress to higher grades through the acquisition of additional alterations, including *CDKN2A* loss and *PDGFRA* or *MET* amplifications, which are associated with poorer prognosis [5]. Like glioblastomas, DLGG are intrinsically heterogeneous at both the molecular and cellular levels. We and others have shown that these tumors contain three main cellular states—astrocyte-like (astrocyte-like), oligodendrocyte-precursor like (OPC-like), and proliferative neural progenitor-like (NPC-like) cells [6–8] which closely resemble their normal brain counterparts and express corresponding markers of astrocytic identity, oligodendroglial lineage, and stemness/proliferation respectively. Notably, only the NPC-like fraction is actively dividing, representing less than 10% of tumor cells, and this proportion increases with tumor grade [7, 8]. In addition, as in many other tumors, gliomas display heterogeneity in cell cycle dynamics, with subsets of cells remaining quiescent while others are actively engaged in various stages of the cell cycle [9]. Several studies have highlighted that quiescent tumor cells are more resistant to therapy and likely contribute to glioma recurrence following treatment [10].

The study of IDH-mutant astrocytomas has long been constrained by the scarcity of relevant in vitro models, limiting our ability to investigate their biological features and malignant progression mechanisms. To date, only a few astrocytoma lines have been reported and well characterized [11, 12], underscoring the need for new experimental systems that faithfully recapitulate these tumors. To address this gap, in this study, we established and thoroughly characterized four novel cell lines derived from IDH-mutant astrocytomas of different grades to provide cellular tools for mechanistic and translational research. We sought to determine whether astrocyte-like, oligodendrocyte-like, and NPC-like states are maintained within these cultures, to explore their relationships with quiescence and plasticity, and to identify some molecular determinants governing state transitions. Furthermore, we sought to find markers to purify astrocyte-like cells, then describe their properties. Together, these models offer valuable tools and molecular resources to investigate lineage dynamics, cellular heterogeneity, and the mechanisms driving tumor progression in IDH-mutant astrocytomas.

## Material and methods

Supplemental material (raw WB together with supplemental figures with legends) and methods can be found in supplemental data 1, 2, 3. Product reference list, concentrations and viruses used can be found in Table S1, S3, S5. 

### Patient-derived cell lines

IDH-mutant astrocytoma samples (Table S2) were obtained from the Neurology fresh tissue collection of the Montpellier University Hospital Biological Resources Center (Gui de Chauliac, CRB@chu-montpellier.fr; Project No. RECH/P722/1-5) with informed consent. Tumors were classified by a neuropathologist according to WHO 2016 and 2021 criteria [13, 14]. Mutation burden was assessed using NGS (or the Sanger method for patient 275), along with IHC for IDH1 R132H, ATRX, and P53 to confirm the molecular profiling of the initial tumors. Patient-derived astrocytoma cell lines (LGG275, LGG85, LGG336, LGG349) were established in defined media as previously described [6, 15, 16]. All experiments were conducted using cells between passages 10 and 15. Glioblastoma cell lines (Gb4, Gb7, Gb21) were generated in-house [17], while oligodendroglioma lines (BT138, BT088) were obtained from collaborators [18]. TG1N and TG20 cells were used as telomerase-positive and ALT-positive controls, respectively, and maintained as previously described [19, 20].

### Cell culture and counting

Cells are cultured in DMEM-F12 supplemented with N2, B27, L-glutamine, and antibiotics. EGF (10 ng/ml), FGF2 (10 ng/ml) and Heparin (2 μg/ml) (Table S1) are added to promote proliferation, while their removal promotes quiescence and differentiation. Cells are dissociated using 0.25% Trypsin–EDTA, followed by inhibition with trypsin inhibitor, CaCl_2_, and DNaseI [6]. For immunofluorescence, cells are seeded on poly-D-lysine and laminin–coated supports at 30,000 cells/cm^2^. For growth assays, cells are plated at 20,000 cells/cm^2^ either on coated surfaces or in polyHEMA-treated wells to promote gliosphere formation. Cells are collected at days 1, 3, 7, and 9, and counted using an automatic BD Z2 cell counter.

### Intracerebral grafting of cell lines

All animal procedures were performed by the Glioblastoma drug evaluation service (Gliotex, ICM, Paris) under procedure 17,503. For orthotopic models, astrocytoma cells were transduced with a virus containing the luciferase gene [21] and implanted into the right caudate-putamen nucleus (Injection Coordinates AP: + 0.1 cm; ML:  − 0.15 cm; DV:  − 0.25 cm) of female athymic Nude mice (Reference Envigo: Hsd: Athymic Nude-Foxn1nu). Luciferase intensity was monitored to analyze tumor progression. Bioluminescence acquisition was performed using the IVIS spectrum Xenogen, and imaged were analyzed using Living Image® acquisition software. Mice were sacrificed and the brain was extracted and frozen at  − 80 °C immediately after sacrifice. Brain sections of 14 μm thickness were made using a Cryostat Microm NX70.

### Immunofluorescence (IF) and histological analysis of mouse brains, and cell culture

Cultured cells were fixed in 4% paraformaldehyde (PFA), permeabilized and blocked (0.1% Triton X-100, 5% donkey serum), and incubated overnight with primary antibodies (Table S3), followed by Alexa Fluor 488/594/647 secondary antibodies for 1 h. Nuclei were stained with Hoechst 33342. Coverslips were mounted with Fluoromount. Images were acquired using a Zeiss Apotome Z2 and analyzed with ImageJ; ≥ 500 cells were counted to quantify cell subpopulations. Mouse brains sections were fixed with PFA 4% then dry at 4 °C. Fixed tissues were permeabilized and blocked (10% BSA, 0.3% Triton X-100). To identify human grafted cells, sections were incubated overnight with a primary antibody human-specific α-NESTIN (Table S3) (PBS 1% BSA, 0.1% triton) followed by Alexa 488 secondary antibody for 1 h. Nuclei were stained with Hoechst 33342. Slides were then mounted with Fluoromount-G. Sections were scanned and acquired using the Leica DM6B Thunder.

### Single-cell 3’ RNA sequencing

#### Cell preparation

To form neurospheres (growth condition), cells were seeded at a density of 200,000 cells/cm² in flasks pre-coated with poly-HEMA in the presence of growth factors. For the differentiation condition, dissociated cells were seeded at 40,000 cells/cm² in flasks coated with poly-D-lysine (PDL) and laminin, in medium devoid of growth factors and heparin. After 4 days of culture, cells were harvested by trypsin/EDTA dissociation, filtered through a 40 µm cell strainer, and pelleted by centrifugation. The pellet was resuspended in 15% Percoll and centrifuged for 5 min at 1,300 rpm and 4 °C to remove debris. Cells were then resuspended in PBS at 1,000 cells/µL for single-cell capture.

#### Library preparation and sequencing

Cell suspensions were loaded onto a Chromium controller (10 × Genomics, Pleasanton, CA) to generate single-cell Gel Beads-in-Emulsion (GEMs). Single-cell RNA-Sequencing libraries were prepared using the Chromium Next GEM Single Cell 3’ V3.1 reagent kits (10X Genomic), according to manufacturer’s protocol. Libraries were denatured with NaOH, neutralized with Tris–HCl, and diluted to 150 pM. Clustering and sequencing were performed on a NovaSeq 6000 (Illumina, San Diego, CA, USA) using the paired-end 28–90 nt protocol on an SP and S4 flow cell lane.

#### Alignment, processing, and quality control of Sc-RNA-sequencing data

Preprocessing of single-cell RNA sequencing (scRNA-sequencing) data was performed using the 10 × Genomics Cell Ranger v7.1.0 pipeline. Raw sequencing datas were processed with “cellranger mkfastq”, followed by “cellranger count” for alignment, filtering, and Unique Molecular Identifier (UMI) counting against the human genome GRCh38-p13. The resulting count matrices were imported into R (v4.4.1) using the Read10X function from the Seurat R package (v5.2.0) [22]. Seurat objects were created for each sample. To remove doublets, a two-step quality control cutoff was applied, which involved the number of unique mRNA/cell (UMI), the number of genes/cell, and the percentage of mitochondrial RNA in the cell. Cells with more than 60,000 UMI/cell, more than 9000 genes/cell or less than 200 genes/cells were removed from the analysis. Cells containing more than 20% of mitochondrial reads were also removed, in accordance with published recommendations for cultured cell lines [23]. This threshold was determined by visual inspection of the distribution of mitochondrial read proportions across all barcodes, selecting a threshold value that preserves viable cells while eliminating outliers. We also used scDblFinder to eliminate doublets. Gene expression was normalized using the LogNormalize method, and the 2000 most variable genes were identified using the FindVariableFeatures function. Principal component analysis (PCA) was performed on these variable genes, followed by UMAP dimensionality reduction using the top 30 principal components. Clustering was performed using the FindNeighbors and FindClusters functions. Downstream analysis was performed independently by sample, each sample as a distinct biological unit thus eliminating inter-sample technical variability and a need of batch effect correction.

#### Analysis of external patient scRNA-sequencing data

Single-nucleus (snRNA-sequencing) and single-cell RNA sequencing (scRNA-sequencing) datasets from IDH-mutant grade 2/3 astrocytomas were retrieved from three independent datasets: two from the Gene Expression Omnibus (GEO; GSE205771 and GSE89567) [7, 24], and a third from a recently published study [25]. Processing, quality control and exclusion of non-malignant cells were performed following scripts provided by the authors of the 3 studies [7, 24, 25]. Exclusion of non-malignant cells were also performed in accordance of the scripts provided by the authors.

#### Annotation

Cell type annotation was performed using a multi-step approach combining the expression of known marker genes, transcription factor expression analysis, co-expression motifs, and ScType [26]. In a first step, the expression of lineage-specific markers for oligodendrocytes and OPCs, astrocytes, and NPCs was analyzed using Seurat’s FeaturePlots and ViolinPlots. Human gene lists for OPC, oligodendrocytes and astrocytes were then collected from CellMarker 2.0 (Copyright© College of Bioinformatics Science and Technology, Harbin Medical University) [27]. The lists were then manually curated to keep only cell type-specific genes checked on The Human Protein Atlas database (Version 23.0 –https://www.proteinatlas.org/). The NPC-like list was constructed from the list established by Zhong et al. [28] and supplemented by the list from Tirosh et al. [8] (Table S4). Gene list score was calculated and used to classify cells into 4 categories: astrocyte-like, oligodendrocyte-like, NPC-like, and unknown (cells with a ScType score < 0). The proportion of annotated cells for each putative cell type was quantified and visualized using pie charts.

For the snRNA-seq dataset of grade 2/3 astrocytoma patients published by Blanco-Carmona et al., cellular state annotation was performed using Non-Negative Matrix Factorization (NMF), as described in the original study [24]. Throughout this manuscript, the states and gene signatures defined in that study are referred to as *NMF states* and *NMF sign.*

#### Differential gene expression analysis and markers validation

To validate cell type annotations and identify robust transcriptional signatures, differentially expressed genes (DEG) were identified with stringent thresholds requiring expression in ≥ 25% of cells within the target population. MAST [29] was used to perform statistical test. DEG were stratified by ScType annotation into four categories: Astrocyte‑like, Oligodendrocyte‑like, NPC‑like, and Unknown cells. For each population, the top 5 most significantly upregulated genes and curated genes from the astrocyte lineage (*ALDOC, APOE, AQP4, CD44, CRYAB, GABBR2, KCNN3, LPL, SLC1A3*), oligodendrocyte lineage (A*SCL1, COL20A1, DLL1, DLL3, EGFR, OLIG1, OLIG2, OPCML, PDGFRA*) NPC lineage (*AURKB, BUB1, E2F7, E2F8, EZH2, FOXM1, MKI67, PCNA*) were prioritized in dotplot visualizations.

To detect similarities in the NMF signatures described by Blanco-Carnoma [24] and our in-house signatures, the top 50 significant marker genes of LGG275 were scored in primary tumor cells from Blanco-Carnoma using Seurat’s AddModuleScore function. Average scores per NMF state were then compared across all our in-house signatures and visualized as a z-scored heatmap.

#### RNA velocity and cellular trajectory analysis

To infer cellular dynamics and differentiation trajectories, RNA velocity analysis was performed with the velocyto and scVelo packages (v0.3.3—^©^Copyright 2024, Theislab) [30]. Preprocessing was performed, then scVelo’s were applied dynamic model to learn the full transcriptional dynamics of splicing kinetics. RNA velocities were estimated and projected onto UMAP. To reconstruct lineage relationships and differentiation trajectories, partition-based graph abstraction (PAGA) was implemented using Scanpy’s tl.paga function. Edge weights in the PAGA graph represent connectivity confidence, indicating potential differentiation paths. Diffusion pseudotime (DPT) was used to infer a pseudotemporal order of cells along predicted routes, defining a root cell within the starting population. Finally, PAGA was extended with velocity-inferred directionality, providing a graphical map of data topology with weighted edges corresponding to cluster connectivity.

#### Cell cycle

Cell cycle analysis was performed using the ccAFv2 algorithm [31]. The default parameter of this classifier was applied to the Seurat object of all samples. To evaluate the correlation between cell phenotype and cell cycle phase as well as frequency, Pearson residuals were calculated and p.value were determined by a Chi2 test.

#### Intercellular communication

To elucidate intercellular communication patterns within LGG275 cells, CellChat v2.1.2 was used [32] with the CellChatDB Human database for ligand-receptor interactions. The probability of intercellular communication was inferred using a mass action model. Communication networks were calculated at both individual ligand-receptor pair and signaling pathway levels. Aggregated intercellular communication network were visualized using chord diagrams. Heatmaps were generated to identify signals contributing most to outgoing or incoming signaling of specific cell types. To discover coordinated communication patterns, matrix factorization was performed on outgoing and incoming intercellular communication probabilities.

### Flow cytometry and cell sorting

After 5 days ± growth factors (GF), cells were dissociated (Accumax or 0.05% trypsin–EDTA for sorting). Surface staining was performed on ice with conjugated antibodies (Table S3). For intracellular staining, cells were fixed (4% PFA), permeabilized (0.1% saponin, 0.5% BSA), and incubated with primary and Alexa 488/647 secondary antibodies, with Hoechst 33342. Acquisition was performed on a MacsQuant (Miltenyi) and analyzed using FlowJo. Live cells were gated using Sytox Blue. Sorting was performed on a FACSAria, and cells were replated at 30,000 cells/cm^2^ ± GF.

### Proteome analysis by mass spectrometry

#### Sample preparation

Protein digestion of the CD44⁺GLAST⁺ and CD44⁻GLAST⁻ subpopulations isolated from LGG275 was performed on S-Trap™ micro columns according to the manufacturer's instructions. 

#### Mass spectrometry

Experiments were performed on a nano-flow HPLC coupled to a mass spectrometer equipped with a nanoelectrospray source. Peptide samples were solubilized in 0.05% trifluoroacetic acid (TFA)/2% ACN, and were injected for desalting and pre-concentration on a PepMap®100 C18 precolumn. Peptide’s separation was done on a analytical reversed-phase column gradient of 2 to 40% of 80% ACN, 0.1% formic acid. Spectra were recorded using Xcalibur 4.2 software. MS analyses were performed in data-independent mode (DIA) using 49 windows (13.7 Th) covering a mass range of 361–1033 m/z. Resolution was set to 120,000 for MS1 and 15,000 for MS2. NCE was set to 27%.

#### Data analysis

Spectral data were analyzed via DIANN v1.8.2b27 software [33]], using the DIAgui v1.4.2 tool [34]]. For protein databases, we used the Homo sapiens Reference Proteome (UP000005640; release 2025_01; https://www.uniprot.org/) and a homemade contaminant database with the following fixed modification: Carbamidomethylation^(C)^. Data reprocessing, statistical analyses and graphical representations were performed using Perseus v1.6.15.0 software [35].

The mass spectrometry proteomics data have been uploaded to the ProteomeXchange Consortium via the PRIDE partner repository with the dataset identifier PXD064235 [36].

### Statistical analyses

Experiments were performed in ≥ 3 independent replicates unless otherwise stated. Data are presented as mean ± SEM. Statistical analyses were conducted using GraphPad Prism v10.4.2 (paired tests), with significance assessed by Student’s *t*-test or one-way ANOVA followed by multiple comparisons (Dunnett or Tukey), as indicated in figure legends.

### Language editing assistance

Stylistic improvements and clarity enhancements were assisted by ChatGPT (OpenAI) artificial intelligence–based language model, and by the corrector Antidote 12 (^©^2025 Druide informatique inc.—v2.0.1).

## Results

### Characterization of 4 cell lines isolated from IDH-mutant astrocytomas

**Fig. 1:**
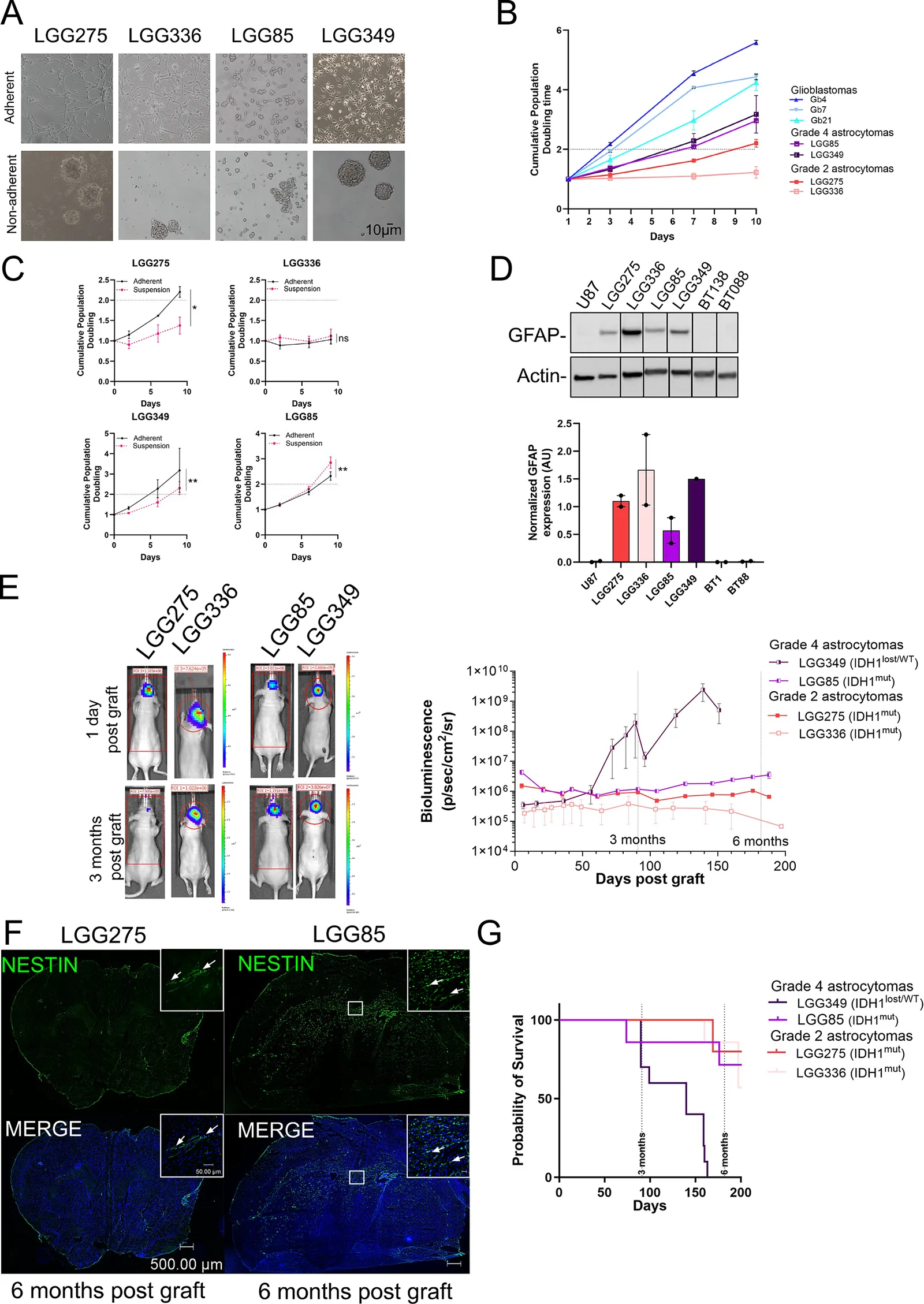
Grade 4 cell lines are more proliferative and tumorigenic than grade 2 cell lines. **A** Cell morphology of the 4 astrocytomas cell lines, in adherent or non-adherent (suspension) conditions **B** Cell doubling time of astrocytoma (n = 3) and glioblastoma (n = 1) cell lines in adherent conditions **C** Comparison of cell doubling time in adherent or non-adherent conditions (n = 3) with GF. Each point represents mean ± S.E.M (ns = non significant, *p< 0,05 et **p< 0,01), as determined by a Student t-test **D** Expression and quantification (arbitrary unit) by western blot of GFAP in astrocytoma cell lines with growth factors (n=2). **E** Bioluminescence visualization and quantification of astrocytoma cell lines grafted in mice (n = 7) **F** hNESTIN expression in astrocytoma cells (LGG275 and LGG85) in mice brain section 6 months post-graft. Nuclei are stained with Hoechst 33342. Arrows indicate hNESTIN+ tumoral cells. Scale bars: 500 µm, and 50 µm for insets.. **G** Kaplan–Meier survival curves of mice grafted with LGG275, 336, 85 or 349 cells, during 6 month post-injection (n = 7)

Using explant cultures maintained in defined medium, we successfully established four adherent cell lines from patients diagnosed with IDH-mutant astrocytomas (Fig. 1A, Table S2). Cell lines were considered established upon successful maintenance beyond 10 passages [6, 15, 16]. LGG275 and LGG336 were derived from histologically classified grade 2 astrocytomas, whereas LGG85 and LGG349 originated from grade 4 astrocytomas. Clinical and molecular data of the source tumors are provided in Table S2. The LGG275 patient’s molecular analysis showed a hemizygous *CDKN2A* deletion, suggesting a higher-grade molecular profile despite its histological grade 2 classification. Cell proliferation assay revealed that LGG85 and LGG349 doubled every 6 days, LGG275 every 9 days, and LGG336 in more than 10 days. In comparison, three previously Gb-derived cell lines [17] proliferated significantly faster, with doubling times of approximately 2–3 days (Fig. 1B).

Given that glioma cell lines are frequently maintained as neurospheres under non-adherent conditions [37–39], we evaluated this alternative culture format. LGG275, LGG336, and LGG349 formed compact neurospheres, whereas LGG85 generated loose cellular aggregates (Fig. 1A). Comparative growth analysis demonstrated that LGG275 and LGG349 proliferated more efficiently under adherent conditions, LGG85 favored aggregate growth, and LGG336 exhibited slow growth in both culture formats (Fig. 1C). To ensure experimental consistency, all subsequent analyses were performed using adherent culture conditions. Western blot (WB) confirmed GFAP expression in all four lines, unlike oligodendroglioma cell lines (BT138, BT088) [37], supporting their astrocytic identity (Fig. 1D).

We next assessed the tumorigenic potential of these lines by intracranial grafting in mice, monitored over 6 months to allow sufficient time for tumor development and enable comparison with previous study on similar IDH1-mutant cultures [12]. Bioluminescence monitoring revealed no detectable tumor growth for LGG275 and LGG336 (Fig. 1E). This was confirmed for LGG275 by histological analysis using a human-specific NESTIN antibody, showing that grafted cells remained confined to the injection site without evidence of proliferation or invasion (Fig. 1F). In contrast, LGG85 and LGG349, derived from higher-grade tumors, exhibited bioluminescent growth, consistent with their more aggressive clinical behavior (Fig. 1E). Histological analysis of LGG85 grafts revealed extensive brain infiltration, with a markedly larger invaded area than LGG275 (Fig. 1F, Fig. S1A). Kaplan–Meier survival analysis further showed that LGG349 was the most aggressive, followed by LGG85, whereas LGG275 and LGG336 had minimal impact on survival (Fig. 1G).

**Fig. 2:**
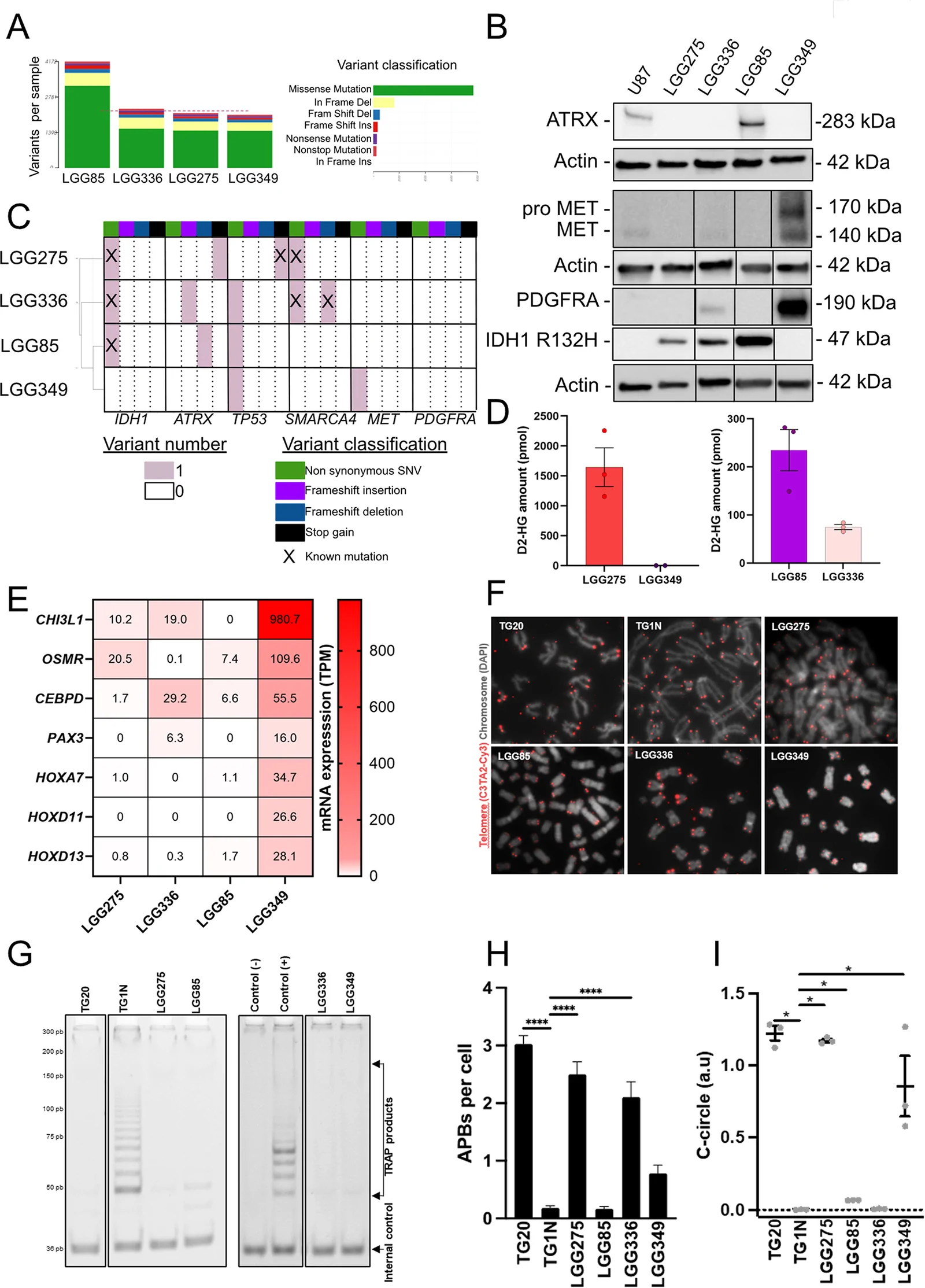
Molecular characteristics of astrocytoma cell lines. **A** Number of genetic variants in astrocytoma cell lines **B** Expression of ATRX, IDH1 R132H, proMET and MET and PDGFRA in astrocytoma cell lines by western blot **C** Number and classification of genetic variants in *ATRX, IDH1, MET, PDGFRA* and *TP53* genes in astrocytoma cell lines after WES using VarDecrypt **D** Intracellular production of 2-HG in astrocytoma cell lines (n = 3). **E** Heatmap of mRNA expression (RNA seq) of mesenchymal (*CHI3L1, OSMR, CEBPD*) and neurodevelopmental (*HOX, PAX)* genes in cell lines, n = 1 **F** Representative chromosomes hybridized with a telomeric PNA probe (red) in Telo-FISH experiments. Chromosomes were counterstained with DAPI (grey) **G** Telomerase activity determined by TRAP and PAGE **H** APBs per cell. Bars show the mean ± S.E.M (**** *p* < 0.001, one-way-ANOVA followed by Dunnett’s multiple comparison test). **I** Detection of C-circles by C-circle assay. Bars show means ± S.E.M, with dots representing individual experimental values (n = 3) (* *p*< 0.05, Mann–Whitney test). Telomerase-positive cells (TG1N) and ALT-positive cells (TG20) were used as controls in all experiments

To comprehensively characterize these lines at the molecular level, we performed whole-exome sequencing (WES) and WB [40]. WES revealed that LGG85 harbored the highest mutational burden (4172 mutations, predominantly missense variants), consistent with its prior temozolomide exposure, which is known to promote hypermutated phenotypes in gliomas (Fig. 2A) [41]. Both WES and WB validated IDH1-R132H mutations in three lines (LGG275, LGG336, and LGG85), while LGG349 has lost it; a phenomenon previously documented during line establishment from IDH-mutant gliomas (Fig. 2B, C) [37, 42]. Consistently, variant allele frequency (VAF) analysis of IDH1 c.395G > A demonstrated robust preservation of the mutation in LGG85 and LGG336, as well as in the LGG275 cell line, even after more than 10 passages, while confirming its loss in LGG349 (Fig. S1B).

Functional validation confirmed 2-hydroxyglutarate (2-HG) secretion in the three *IDH1*-mutant lines but not in LGG349, corroborating the genomic findings (Fig. 2D). As anticipated for astrocytic tumors, *TP53* mutations were present across all four lines, while *ATRX* alterations occurred in three lines (Fig. 2C). ATRX protein was absent in LGG275, LGG336, and LGG349 but retained in LGG85 and the Gb line U87 (positive control) (Fig. 2B). Remarkably, both LGG275 and LGG336 carried mutations in *SMARCA4*, encoding a chromatin remodeling complex component (Fig. 2C) [5].

Consistent with its aggressiveness in vivo behavior, LGG349 displayed molecular features characteristic of high-grade astrocytoma, including *MET* mutations and overexpression of both MET and PDGFRA (Fig. 2B and C, Fig. S1C, D). Importantly, high-MET expression was already detectable by IF in the original patient’s tumor, confirming this alteration preceded culture adaptation (Fig. S1E). RNA-sequencing (Fig. 2E) further revealed specific expression of mesenchymal markers (*CHI3L1, OSMR, CEBPD*) and neurodevelopmental transcription factors (*HOXA7, HOXD11, HOXD13, PAX3*) in LGG349, consistent with activation of HOX/neurodevelopmental programs during malignant progression of IDH-mutant gliomas [43–46].

### Evidence of alternative lengthening of telomeres in astrocytoma cell lines

*ATRX* is a chromatin remodeler that maintains heterochromatin stability at telomeres and repetitive regions. Its loss is associated with alternative lengthening of telomeres (ALT), a telomerase-independent mechanism that extends telomeres via homologous recombination. ALT occurs in about 23% of astrocytomas [47], but there are limited in vitro models for this process in these glioma subtypes [48]. ALT features in our cell lines were assessed by examining telomere length heterogeneity, telomerase activity, ALT-associated PML bodies (APBs), and c-circles [49–52], with TG1N and TG20 Gb used as negative and positive controls, respectively [20]. Telomere-FISH revealed heterogeneous telomere lengths, ranging from undetectable or small signals to ultra-bright foci across our four lines, in contrast to telomerase-positive TG1N cells showing homogeneous telomere foci (Fig. 2F). TRAP assays showed that telomerase activity was undetectable in LGG275, LGG336, and LGG349, and low in LGG85 (Fig. 2G). IF of PML proteins combined with Telomere-FISH revealed ALT-associated PML nuclear bodies (APBs) in LGG275 and LGG336, but not in LGG85 or LGG349 (Fig. 2H). Moreover, telomeric c-circles were detected in LGG275, LGG85, and LGG349 (Fig. 2I). Together, these findings indicate that only LGG275 exhibits all canonical ALT features, whereas the other lines show partial features, suggesting heterogeneous telomere maintenance or incomplete ALT activation.

### Astrocytoma cell lines retain astrocyte-like, oligodendrocyte-like, and NPC-like cell states

**Fig. 3:**
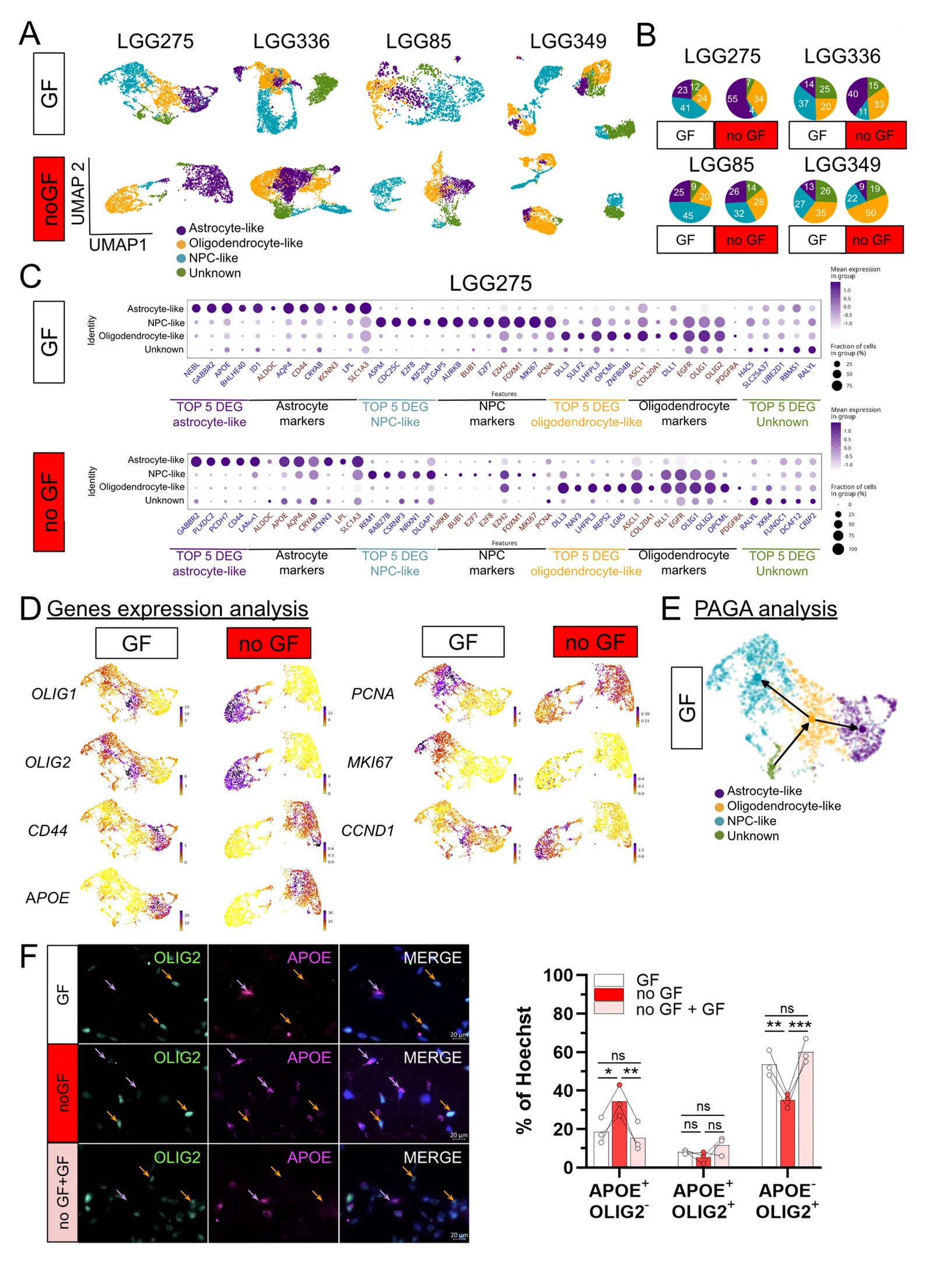
Astrocytoma cell lines exhibit multiple cellular states, reflecting the heterogeneity observed in patient tumors.** A** UMAP visualization of scRNA-seq transcriptomic clusters from four astrocytoma cell lines cultured with (GF) or without (no GF) growth factors. **B** Cellular state proportions in astrocytoma cell lines with or without GF (**C**) DotPlot of curated differentially expressed genes (DEG) (TOP5 + markers of astrocytes, oligodendrocytes or NPC) in each cellular state in LGG275 cell line with or without growth factors. Genes in blue are among the TOP20 DEG **D** Examples of oligodendrocyte-like (*OLIG1, OLIG2*) and astrocyte-like specific (*APOE, CD44*) gene expression measured by scRNA-seq in distinct cell subpopulations of the LGG275 cell line cultured with or without GF. Genes are differentially expressed between each subpopulation with a *p*.value < 0.01 as determined by a Wilcoxon test **E** Partition-based graph abstraction (PAGA) analysis integrating RNA velocity to infer trajectories between cell subpopulations **F** Immunofluorescence (IF) analysis of APOE and OLIG2 expression in the LGG275 cell line, identifying “astrocyte-like” APOE⁺OLIG2⁻ cells (purple arrows) and “oligodendrocyte/NPC-like” APOE⁻OLIG2⁺ cells (orange arrows) under different growth factor conditions: with (GF) or without GF (no GF) for 5 days, or without GF for 5 days followed by GF re-exposure for 5 days (no GF + GF). Right panel: Barplot shows quantification as % of Hoechst ⁺ cells. Mean (bars) and individual values (dots) from n = 3 independent experiments. (*p <  0.05, **p < 0.01, **p <  0.001, multiple comparisons Tukey test)

To determine whether our cell lines recapitulate the cellular heterogeneity of IDH-mutant astrocytomas [6–8, 24], we performed single-cell RNA sequencing in the presence of growth factors. Annotation using our in-house cell type signature set (Table S4) identified three major cellular states; astrocyte-like, oligodendrocyte-like, and NPC-like, consistently observed across all lines (Fig. 3A, B). To further validate these annotations, we performed Gene Set Enrichment Analysis (GSEA) on cluster-specific genes. In LGG275 (Fig. S2A) and across all other lines (Table S6), GSEA confirmed robust enrichment of astrocyte-like, oligodendrocyte-like, and NPC-like transcriptional programs. Typical markers for proliferation/NPC (e.g., *AURKB, E2F7*), astrocytes (e.g., *APOE, AQP4, CD44, CRYAB, GABBR2, ID1*), and oligodendrocytes (e.g., *DLL3, OLIG1, OLIG2, OPCML*) were predominantly expressed in the corresponding clusters (Fig. 3C). Proliferative genes were absent from astro- and oligodendrocyte-like states (Fig. 3C and D). Additionally, “unknown” cells were detected in all lines, with higher proportions in LGG336 and LGG349, suggesting subclonal heterogeneity (Fig. 3A).

Overlap between significant cluster DEG (dark blue) and the astrocyte-like, oligodendrocyte-like, and NPC-like reference gene sets (light blue) was similar in LGG275, LGG336, and LGG349, but substantially lower in LGG85 under GF conditions (Fig. S2B, Table S7). Compared to LGG275, cell-type boundaries in other lines were also less distinct. For example, in LGG336 and LGG349, NPC-associated genes (*EZH2, FOXM1*) were strongly found in astrocyte- and oligodendrocyte-like clusters, while oligodendroglial markers (*OLIG1, OLIG2*) were present in astrocyte-like clusters. Astrocytic markers (*CD44, CRYAB, GABBR2, KCNN3*) were enriched in astrocyte-like clusters but extended to others (Fig. S2C, D). LGG85 expressed them minimally (Fig. S2E). These results are consistent with the idea that increased mutational burden may be associated with weaker lineage signature coherence in favor of cell cycle signatures [53].

We also assessed cellular state proportions after 5 days of growth factor withdrawal (noGF), which reduced proliferation across all four lines, as shown by RNA-seq and GSEA (Fig. S2F, G). Remarkably, while the NPC-like population dropped sharply by ~ tenfold (41 → 4%) in LGG275 and ~ 3.3-fold (37 → 11%) in LGG336, this decline was much less pronounced in the two high-grade astrocytoma-derived lines, with only a ~ 1.4-fold reduction in LGG85 (45 → 32%) and a ~ 1.2-fold reduction in LGG349 (27 → 22%) (Fig. 3B). These findings suggest that, as astrocytomas progress toward higher malignancy, the NPC-like state expands and becomes less dependent on exogenous growth factors for maintenance [54].

To assess the relevance of the cellular states identified in our cell lines with respect to primary tumors, we projected the gene signatures used to define our clusters onto an independent snRNA-sequencing datasets of 6 grade 2/3 IDH-mutant astrocytomas (Blanco-Carmona dataset) [24]. As shown in Fig. S3A, there was a strong correspondence between the oligodendrocyte-like, astrocyte-like, and NPC-like clusters defined by our signatures and those identified in this study. In this cohort, the authors also described gradient and ribosome-enriched (RE) subclusters within the astrocyte-like and oligodendrocyte-like populations, which correspond to the astrocyte-like and oligo/NPC-like states defined in our dataset (Fig. S3B). Compared to tumors, likely due to the presence of exogenous GF, NPC-like cells were overrepresented in our cultures; however, upon GF withdrawal, the cellular composition more closely resembled that observed in primary tumors, notably in LGG275. Analysis of the top five differentially expressed genes (DEG) in their clusters, as well as canonical markers of astrocyte-like, oligodendrocyte-like, and NPC populations, showed strong concordance with the signatures used to define our three clusters (Fig. S3C). Consistent results were obtained from the analysis of two additional independent scRNA seq astrocytoma cohorts, further supporting the relevance of the clusters identified in our cell lines to those observed in patient tumors (Fig. S3D-G) [7, 25].

To pursue downstream analyses, LGG275 was prioritized due to its patient-like scRNA-seq architecture, reduction of NPC-like cells upon growth factor withdrawal, and lack of tumorigenicity in vivo. In addition, its canonical IDH-mutant astrocytoma genotype (*ATRX* loss, *TP53* mutation, IDH1-R132H) supports its relevance as a model of slow-growing astrocytoma.

To delineate relationships between cell states in LGG275, we computed a PAGA graph and oriented edges using RNA velocity estimates from scVelo [30]. The resulting graph suggested potential directional transitions from oligodendrocyte-like cells toward NPC-like and astrocyte-like populations (Fig. 3E). While these analyses should be interpreted cautiously, they are consistent with cellular plasticity and suggest that oligodendrocyte-like cells may represent a putative intermediate transcriptional state within a continuous cellular landscape.

To confirm the presence of astrocyte-like and oligodendrocyte/NPC-like subpopulations in the LGG275 line, we used IF with APOE as an astrocyte marker and OLIG2 as an oligodendrocyte/NPC marker (Fig. 3D left, 3F). With GF, 20% of cells are APOE^+^OLIG2^−^, 57% are APOE^−^OLIG2^+^, and ~ 10% co-expressed both. Consistent with the scRNA-sequencing data, GF withdrawal increased the proportion of astrocyte-like (APOE⁺OLIG2^−^) cells to 38% and reduced oligo/NPC-like (APOE^−^OLIG2⁺) cells to 39%. To explore the plasticity of these states, we reintroduced GF (noGF + GF) and found that the proportions of APOE^+^OLIG2^−^ and APOE^−^OLIG2^+^ cells returned to baseline, suggesting reversibility and dynamicity in these state transitions. Similar IF analyses using additional astrocytic markers (GFAP, FABP7) and oligodendrocyte/NPC associated markers (MASH1, OLIG1) confirmed the coexistence of astrocyte-like and oligodendrocyte-like populations in LGG275 cells (Fig. S4A). Flow cytometry with CD44 (astrocyte-like) and OLIG1 (oligodendrocyte/NPC-like) further corroborated these findings (Fig. S4B). In contrast, the other three lines did not display clear clustering, reinforcing that LGG275 better captures the cellular heterogeneity observed in slow-growing astrocytomas (Fig. S4B).

### Astrocyte-like cells display features consistent with a quiescent state in the LGG275 line

**Fig. 4:**
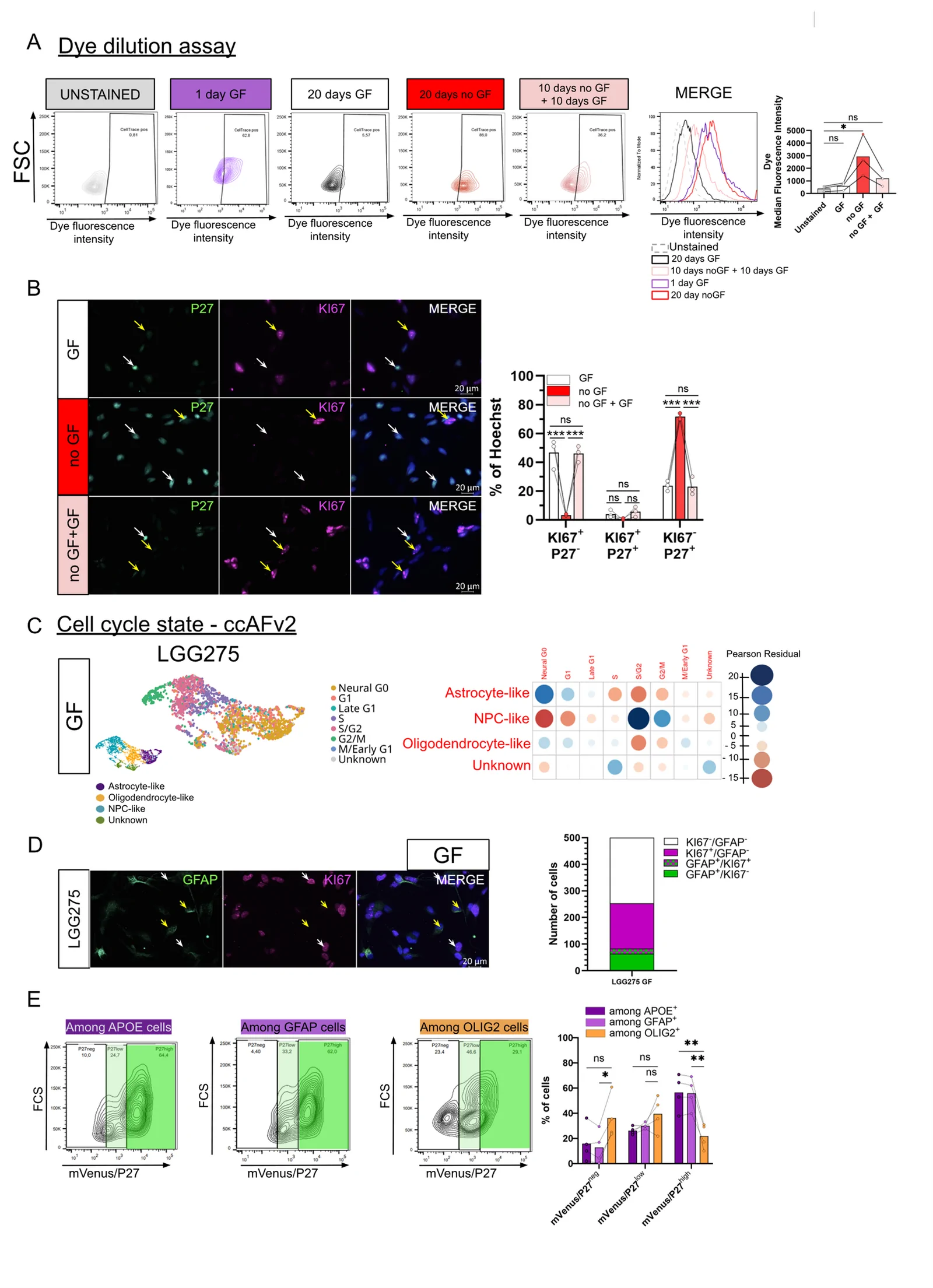
Astrocyte-like cells in the LGG275 cell line are enriched in a quiescent (Neural G0) state.** A** CellTrace dye-dilution assay by flow cytometry in LGG275 lines after 20 days in “GF”, “no GF” and “no GF + GF” conditions. Left: Representative plots (FSC/CellTrace fluorescence) for unstained cells and CellTrace-labeled cells after 1 day GF, 20 days GF, 20 days no GF, or 10 days no GF + 10 days GF. Middle: Overlaid bars (MERGE) show CellTrace fluorescence intensity. Right, CellTrace median fluorescence intensity (MFI) quantification. (mean ± S.E.M; dots = independent experiments, n = 3) (***  *p*< 0,001) as determined by a Mann–Whitney multiple comparisons. **B** KI67/P27 IF in LGG275 glioma cells cultured in GF, no GF, or no GF + GF conditions. Proliferating KI67⁺P27⁻ cells ( yellow arrows) and quiescent KI67⁻P27⁺ cells (white arrows) are indicated. Right, Barplot show quantification as % of Hoechst⁺ cells (bars = mean; dots = n = 3); (***  *p*< 0,001) as determined by one-way ANOVA with Tukey’s multiple comparisons. **C** ScRNA-seq analysis of LGG275 cells cultured in GF. Left, UMAP colored by ccAFv2 cell-cycle state classifier. Right, dot plot showing the association between phenotypic states (astrocyte-like, NPC-like, oligodendrocyte-like, unknown) and ccAFv2 cell-cycle classes (Pearson residual). Significance was assessed by a χ^2^ test (*p* < 0.01). **D** Left: IF for GFAP and KI67 in LGG275 cells grown with GF showing two non-overlapping astrocyte-like (GFAP^+^, yellow arrows) and mitotic (KI67^+^, white arrow) populations (n = 1). Right: Bar plot shows quantification. **E** Flow cytometry analysis of the mVenus/P27 fusion reporter in LGG275 cells. Cells cultured with growth factors (GF) were stained for APOE, GFAP, and OLIG2 and analyzed by FACS to assess reporter expression. Representative density plots show mVenus/P27 fluorescence among APOE⁺, GFAP⁺, and OLIG2⁺ gated cells. Right: Barplot shows quantification of APOE⁺, GFAP⁺, and OLIG2⁺ cell fractions across mVenus/P27 expression levels (bars = mean; dots = n = 3) (ns, not significant; *p < 0.05; **p < 0.01, Tukey’s multiple comparisons test)

It is established that tumors contain subpopulations of cells that are either in a quiescent state or actively proliferating, and this also applies to gliomas [9]. However, the presence of such states in astrocytoma lines remains poorly characterized. To first access cell cycle phase of LGG275 cells, we performed a cell cycle analysis, which revealed that the vast majority of cells were not actively cycling and are in G0/G1 phase (Fig. S5A). To determine whether most LGG275 cells eventually re-enter the cell cycle and actively divide, we carried out dye dilution assays (CellTrace) with or without GF. As shown in Fig. 4A, after 20 days, cells cultured with GF had largely lost their dye fluorescence, whereas those deprived of GF retained it. This indicates that the majority of LGG275 cells entered the cell cycle over this period and that quiescent cells can be driven back into proliferation by GF, supporting the existence of a reversible quiescent state. To visually distinguish between proliferating and quiescent subpopulations in LGG275, we used IF for *CDKN1B*/P27, an indicator of quiescence, and KI67. Figure 4B shows two distinct populations of KI67^−^P27^+^ and KI67^+^P27^−^ cells. As expected, GF withdrawal increased the proportion of KI67^−^P27⁺ (quiescent) cells, while reintroducing GF restored the initial proportions.

To isolate quiescent cells, we engineered LGG275 cells to express a mVenus/P27 fusion protein via viral transduction [55]. Since P27 is rapidly degraded upon cell-cycle entry, fluorescence intensity enabled discrimination between quiescent (mVenus/P27⁺) and cycling (mVenus/P27⁻) cells. GSEA of RNA-sequencing profiles from mVenus/P27^+^ and P27/Venus^−^ fractions showed a marked depletion of proliferation-associated genes in the mVenus/P27^+^ population, confirming reporter specificity (Fig. S5B, Tables S8 and S9). After sorting and culturing mVenus/P27⁺ and mVenus/P27⁻ cells, both populations regenerated similar proportions of each type within 4 days, confirming that quiescence in LGG275 cells is reversible and dynamically regulated (Fig. S5C).

As our scRNA-sequencing analysis revealed several clusters, we next sought to examine the relationship between cell phenotype and proliferative or quiescent state. To this end, we used several complementary approaches. First, we applied a new classifier (**ccAFv2**) [31] providing improved resolution of G0-phase cells compared with Seurat’s *CellCycleScoring* function, which cannot reliably distinguish G0 from G1. This approach identified a population of cells in a neural G0 state within the LGG275 line (Fig. 4C). Notably, this population mostly coincided with the astrocyte-like cluster (Fig. 4C, left). Indeed, analysis of the four cellular states in LGG275 (Fig. 4C, right, Table S10) using this classifier showed that neural G0 (quiescent) gene program was strongly correlated to astrocyte-like phenotype (Pearson residual (PR) = 16.0), then oligodendrocyte-like phenotype (PR = 6.0), and negatively correlated to NPC-like identity (PR = − 14.1). In contrast, proliferative phases (S/G2 and G2/M) were strongly associated with NPC-like phenotype (PR = 21.6 and 12.7), as expected. G1 and M/EarlyG1 programs are linked to oligodendrocyte-like cells (PR = 4.7 and 4.1) suggesting a transient phase for this state.

We experimentally validated these predictions using IF and flow cytometry. Double labeling for GFAP and KI67 revealed that the two populations were largely mutually exclusive in LGG275 cells (Fig. 4D). Triple KI67/APOE/OLIG2 staining revealed that KI67 expression was predominantly associated with APOE^−^OLIG2⁺ cells, rather than APOE⁺OLIG2^−^ cells, with (Fig. 4D right) or without GF (Fig. S6A). Consistently, flow cytometry analysis of the mVenus/P27 reporter line showed that P27^high^ cells were more frequently APOE⁺ and GFAP^+^ than OLIG2⁺ (Fig. 4E), further supporting that astrocyte-like cells preferentially adopt a more quiescent state.

The relationship between cellular identity and quiescent state was also studied in other lines. The ccAFv2 classifier revealed that cells in the neural G0 phase of LGG336 and LGG349 exhibited mixed astrocyte-like and oligodendrocyte-like signatures (Fig. S6B). In the high-grade LGG85 line, which contains only a few astrocyte-like cells, neural G0 cells were instead enriched with oligodendrocyte-like markers. Thus, although the classifier consistently identified quiescent populations across all lines, their lineage identity was more heterogeneous than in LGG275. Nevertheless, GFAP and KI67 IF analyses showed reduced proliferative activity in GFAP^+^ cells, suggesting astrocyte-like characteristics in the quiescent compartment in all lines (Fig. S6C).

### Astrocyte-like and oligodendrocyte-like cells in LGG275 exhibit transcriptional similarities to quiescent and activated neural stem cell programs

**Fig. 5:**
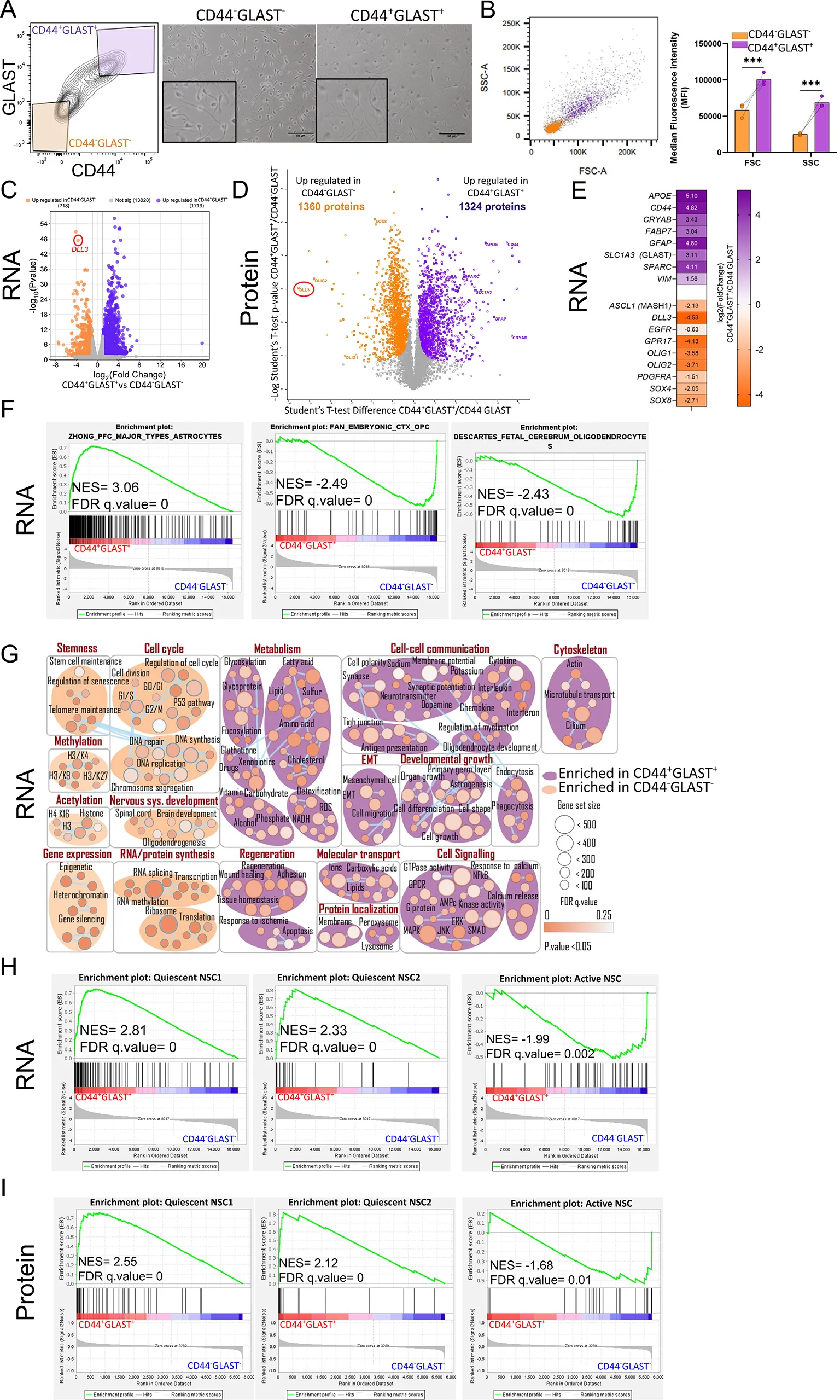
Characterization of astrocyte-like and oligodendrocyte-like populations in LGG275 cells.** A** Morphology of astrocyte-like and oligodendrocyte-like states. Left: representative flow cytometry plot showing gates used to isolate CD44⁻GLAST⁻ and CD44⁺GLAST⁺ cells. Right, phase-contrast images of the corresponding sorted populations; boxed areas indicate higher-magnification views. Scale bar, 50 µm **B** Size and granularity of CD44^+^GLAST^+^ and CD44^−^GLAST^−^ cells analyzed by flow cytometry (Histograms = means; dots = n = 3) (*** *p*< 0.001, Student’s t-test) **C** Volcano plot of DEG between CD44^+^GLAST^+^ and CD44^−^GLAST^−^ cells (RNA-sequencing). Orange and purple dots represent differentially expressed genes with a Log2(FoldChange) > 1 and *p*.value < 0.05 as determined by a Student’s t-test. **D** Volcano plot of differentially expressed proteins between CD44^+^GLAST^+^ and CD44^−^GLAST^−^ populations measured by mass spectrometry. Orange and purple dots represent differentially expressed proteins with a FoldChange > 1 and p-value < 0.05 as determined by a Student’s t-test. **E** Heatmap showing Log2(FoldChange) for selected astrocytic (*APOE, CD44, CRYAB, FABP7, GFAP, SLC1A3, SPARC,* and *VIM*) and oligodendrocyte/NPC-like (*ASCL1*, *DLL3, EGFR, GPR17, OLIG1, OLIG2, PDGFRA, SOX4* and *SOX8*) comparing CD44^+^GLAST^+^ versus CD44^−^GLAST^−^ populations (RNA-sequencing, n = 3). Differential expression was computed with DESeq2; all genes are significant (*p* < 0.01). **F** GSEA of RNA-seq data comparing sorted CD44⁺GLAST⁺ and CD44⁻GLAST⁻ subpopulations (n = 3), showing enrichment of astrocyte and OPC/oligodendrocyte gene signatures in the CD44⁺GLAST⁺ and CD44⁻GLAST⁻ cells, respectively (n = 3). NES = Normalized Enrichment Score, FDR = False Discovery Rate. *p*.value has been calculated using the Benjamini–Hochberg method. **G** Cytoscape visualization of enriched signaling pathways (Gene Ontology) in CD44^+^GLAST^+^ and CD44^−^GLAST^−^ populations from GSEA analysis. *p*-value and q-value were calculated using the Benjamini–Hochberg method. **H** GSEA of RNA-sequencing data comparing CD44⁺GLAST⁺ and CD44⁺GLAST⁺ sub-populations (n = 3) show enrichment of qNSC1/NSC2 signatures in CD44⁺GLAST⁺ cells, and the aNSC signature in CD44⁻GLAST⁻ cells **I** Proteomics-based GSEA comparing CD44⁺GLAST⁺ and CD44⁻GLAST⁻ cells (n = 3) show enrichment of qNSC1/NSC2 signatures in CD44⁺GLAST⁺ cells, and the aNSC signature in CD44⁻GLAST⁻ cells

The clear coexistence of astrocyte-like and oligodendrocyte-like states within the LGG275 line provides a unique model to investigate their respective properties and interactions. ScRNA-sequencing identified CD44 and GLAST (SLC1A3) as markers for astrocyte-like cells (Fig. 3C, D), and we used them to isolate CD44⁺GLAST⁺ and CD44⁻GLAST⁻ populations, after GF withdrawal. CD44⁺GLAST⁺ cells exhibited larger, complex morphologies, while CD44⁻GLAST⁻ cells were smaller and bipolar (representative images, Fig. 5A; flow cytometry of SSC/FSC, Fig. 5B). To deeply characterize these 2 populations, we first performed RNA-sequencing on each sorted subpopulation, identifying 2431 differentially expressed genes (DEG); 1713 enriched in CD44⁺GLAST⁺ and 718 in CD44⁻GLAST⁻ cells (Fig. 5C, Table S11). Extending this analysis at the protein level, quantitative proteomic analysis revealed 1324 proteins enriched in CD44⁺GLAST⁺, and 1360 proteins enriched in CD44⁻GLAST⁻ cells (Fig. 5D, Table S12). As expected, astrocytic markers (APOE, CRYAB, FABP7, GFAP, SPARC, VIM) were enriched in CD44⁺GLAST⁺ cells, while oligodendrocytic markers (ASCL1, DLL3, EGFR, GPR17, OLIG1/2, PDGFRA, SOX4/8) dominated in CD44⁻GLAST⁻ cells (Fig. 5D, E, Table S11). RT-qPCR confirmed their selective expression in both populations (Fig. S7A). GSEA indicated that CD44⁺GLAST⁺ cells were enriched in astrocyte [28] and astrocyte-like [56] gene signatures, whereas CD44⁻GLAST⁻ cells exhibited enrichment in oligodendrocyte [57], OPC [58], and NPC-like [56] programs (Fig. 5F, Fig. S7B).

Integrated Cytoscape visualization of RNA-seq GSEA and proteomic enrichment analyses (Fig. 5G, Fig. S8A, Tables S13 and S14) suggested enrichment of cell communication, metabolism, and signaling pathways in astrocyte-like (CD44⁺GLAST⁺) cells. Developmental pathways, including astrogenesis, were also observed. These cells also expressed genes linked to epithelial/proneural-mesenchymal transition (EMT/PMT), ECM interaction, and migration, consistent with features of a partial mesenchymal phenotype. These metabolic and signaling characteristics are similar to those reported in normal astrocytes [59–64] and in quiescent glioma stem cells (qGSC) from high-grade gliomas [65]. CD44⁻GLAST⁻ cells showed higher expression of genes related to cell cycle, stem cells, telomere maintaining, and epigenetic control, suggesting a more proliferative, NPC-like transcriptional state.

An additional and intriguing observation concerns the relationship between these two populations and the two neural stem cell (NSC) states found in the mouse brain subventricular zone stem cell niche (SVZ). In this niche, NSC transition between quiescent (qNSC) and primed/activated (aNSC) states, with quiescence further subdivided into qNSC1 and qNSC2 sub-states [66–71]. GSEA comparisons with SVZ datasets [68] using both our RNA-sequencing and proteomic data indicated that CD44⁺GLAST⁺ astrocyte-like cells display transcriptional similarities to qNSC, whereas CD44⁻GLAST⁻ oligodendrocyte-like cells show similarities to aNSC (Fig. 5H, I, Fig. S8B). This parallel suggests that the coexistence of these two populations within LGG275 may reflect a reversible continuum between qNSC and activated aNSC-like states.

### Astrocyte-like cells (CD44⁺GLAST⁺) display plasticity and contribute to the restoration of cellular heterogeneity in LGG275 line

**Fig. 6:**
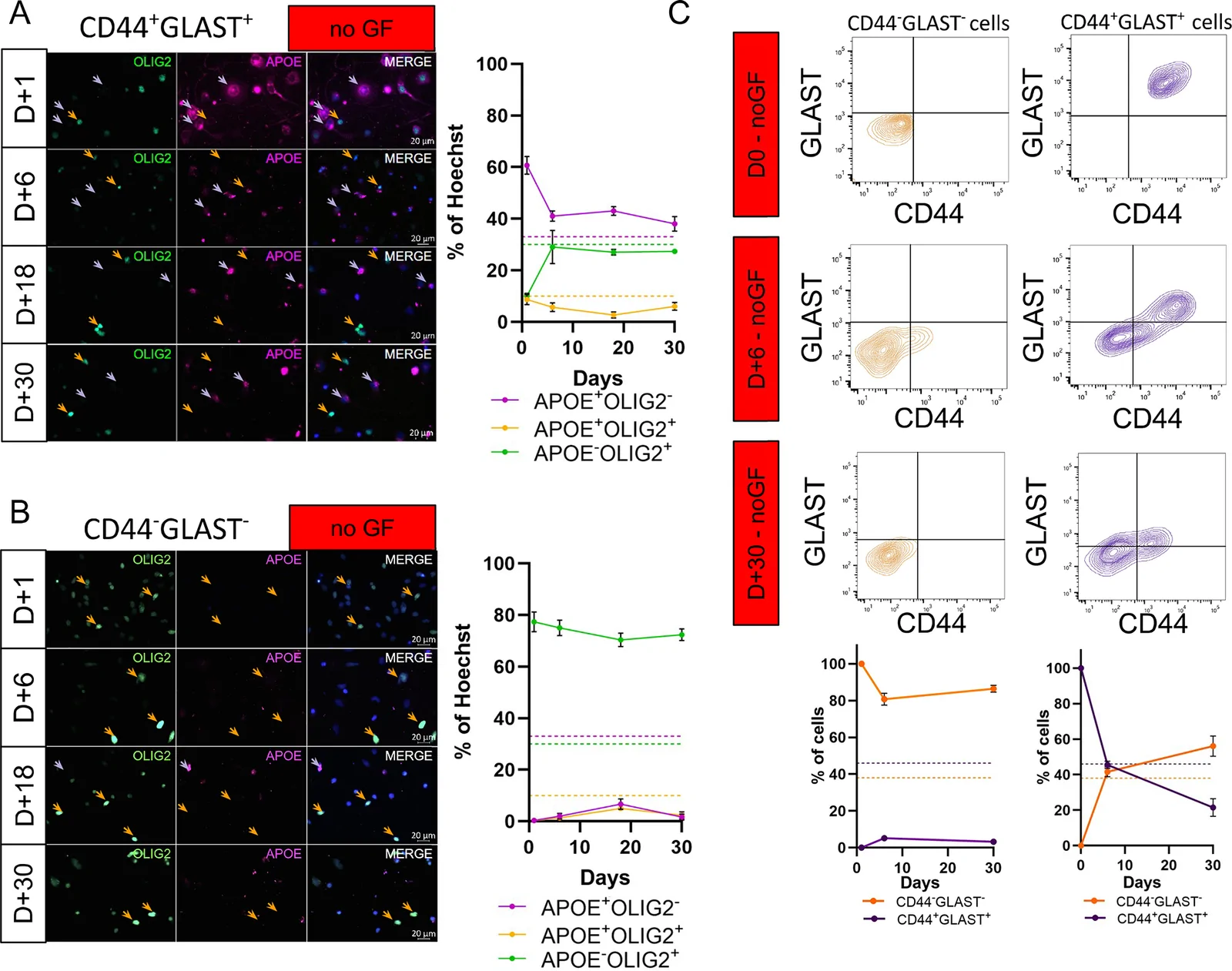
Astrocyte-like cells are plastic and play a role in the cellular heterogeneity formation in the LGG275 line. **A,B **IF analysis of APOE and OLIG2 expression, identifying "astrocyte-like" APOE⁺OLIG2⁻ cells (purple arrows) and "oligodendrocyte/NPC-like" APOE⁻OLIG2⁺ cells (orange arrows) in **A** CD44⁺GLAST⁺ and **B** CD44⁻GLAST⁻ sub-populations purified from LGG275 cells, 1, 6, 18, and 30 days after sorting and plating without GF. Barplot show quantification as % of Hoechst⁺ cells (dots on curve = mean ± S.E.M, n=3) **C** Flow cytometry expression of CD44 and GLAST markers in the LGG275 cell line in CD44+GLAST+ and CD44-GLAST- populations, 6 or 30 days after cell sorting, in the absence of growth factors. Barplot show quantification as % cells (dots on curve = mean ± S.E.M, n=3)

We then assessed the stability and plasticity of astrocyte-like and oligodendrocyte-like states by separately culturing FACS sorted CD44⁺GLAST⁺ and CD44⁻GLAST⁻ populations observed without GF, monitoring APOE and OLIG2 expression over 30 days. As shown in Fig. 6A, the proportion of APOE⁺OLIG2⁻ cells decreased from approximately 60% to 40% , while APOE⁻OLIG2⁺ cells increased from about 5-10% to 25-30% by day 7 in purified CD44⁺GLAST⁺ cells. By day 30, the proportions of the two populations became nearly equivalent. As cell division was minimal without GF (Fig. 3A–C; see *MKI67* in Fig. 3D right), these dynamics are consistent with phenotypic transitions from APOE⁺OLIG2⁻ to APOE⁻OLIG2⁺ states rather than proliferative expansion. Similar trends were observed in the presence of GF (Fig. S9A). We performed the same experiment using CD44⁻GLAST⁻ cells (Figs. 6B, S9B). One day after sorting, 80% of these cells were APOE⁻OLIG2⁺, and their phenotype remained largely stable, with only a slight increase in APOE expression over time. These observations were further supported by FACS-based tracking of CD44 and GLAST expression in the two purified populations. CD44⁺GLAST⁺ cells progressively lost these markers and gave rise to a CD44⁻GLAST⁻ population (Fig. 6C). In contrast, purified CD44⁻GLAST⁻ cells did not reacquire CD44⁺GLAST⁺ markers under these conditions. Together, these findings are consistent with the idea that astrocyte-like cells display a higher degree of phenotypic plasticity and may transition toward oligodendrocyte-like states, thereby contributing to the re-establishment of cellular heterogeneity in the LGG275 line. In contrast, oligodendrocyte-like cells appear more stable and show limited capacity to revert to an astrocyte-like transcriptional state under the conditions tested.

### NOTCH signaling is associated with cellular plasticity and state dynamics in LGG275

**Fig. 7:**
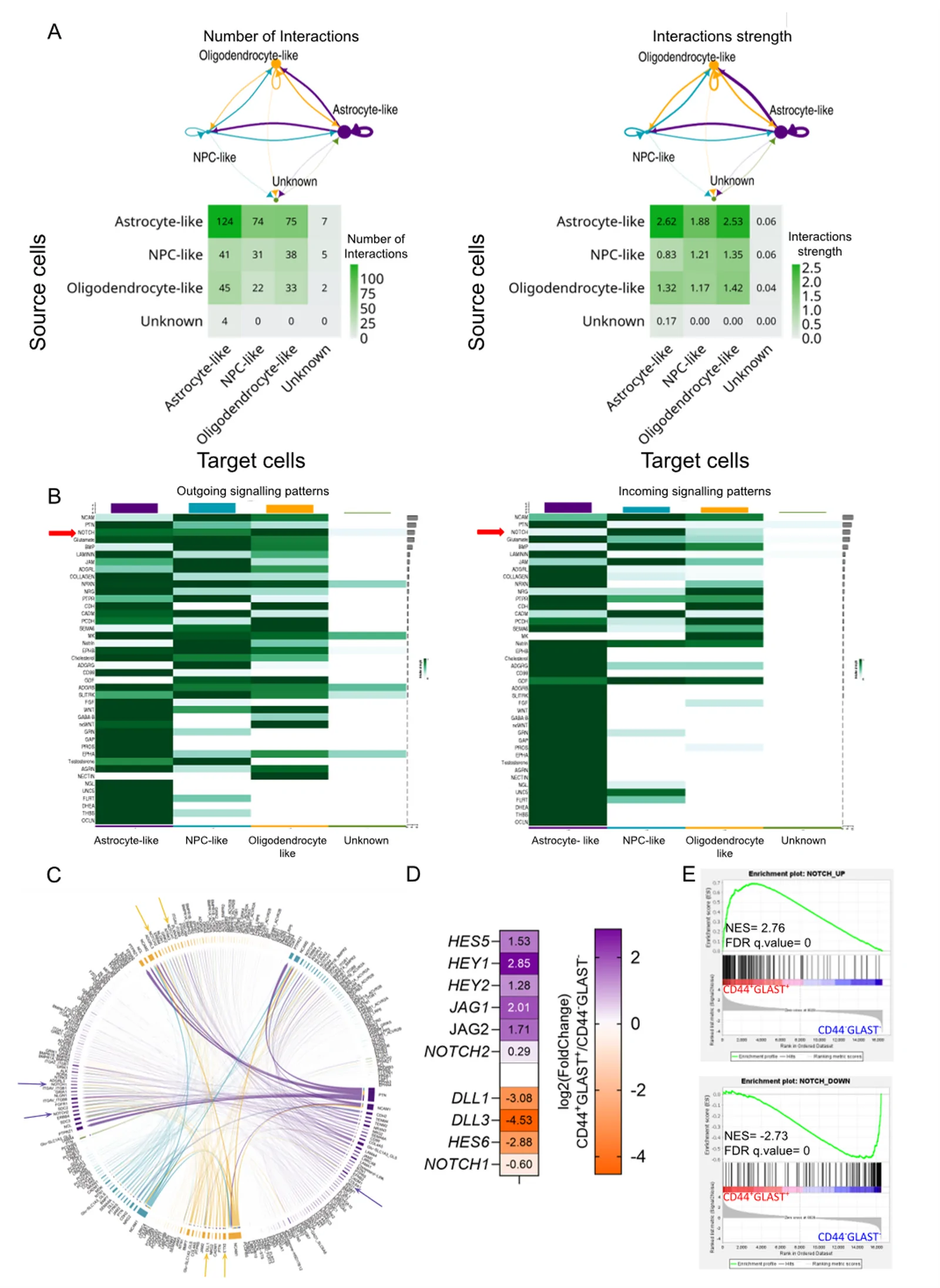
Bioinformatic inference suggests NOTCH pathway involvement in communication between cellular states in the LGG275 line. ** A** String diagrams and heatmaps representing the number and strength of autocrine and paracrine cell interactions in each cell subpopulations, grown without GF. Analysis was performed using the CellChat v2.1.2 tool on scRNA-sequencing cell data **B** CellChat heatmap of outgoing and incoming signaling patterns inferred from scRNA-seq data. Rows indicate ligand–receptor signaling pathways; columns represent the indicated color-coded cell subpopulations. Color intensity denotes the normalized communication strength (darker = stronger). The size of the color-coded top box plots summarize the overall contribution of each subpopulation, and the right bar plot ranks pathways by overall information flow. Notch pathway is indicated by red arrow. **C** CellChat chord diagram showing inferred ligand–receptor communication among cell subpopulations (colors as in prior panels) from scRNA-seq data of LGG275 cells, grown without GF. Chord thickness reflects interaction strength, and outer labels indicate key ligand/receptor genes (arrows highlight NOTCH receptors and ligands). **D** Heatmap of canonical Notch signaling pathway genes differentially expressed between CD44⁺GLAST⁺ and CD44⁻GLAST⁻ cells. Values represent log₂ fold changes (CD44⁺GLAST⁺/CD44⁻GLAST⁻) obtained from differential expression analysis performed using DESeq2 (volcano plot). All genes are significant (*p* < 0.01). **E** GSEA analysis performed in CD44⁺GLAST⁺ and CD44⁻GLAST⁻ populations (n = 3) using NOTCH_UP and NOTCH_DOWN gene sets derived from LGG275 cells following DLL4-mediated NOTCH activation (Table S4) NES = Normalized Enrichment Score, FDR = False Discovery Rate

Given the coexistence of multiple cellular states in LGG275 and the asymmetric plasticity observed between astrocyte-like and oligodendrocyte-like populations, we hypothesized that intercellular signaling regulates these transitions. To predict potential ligand/receptor interactions among LGG275 cells, we applied the CellChat tool on our scRNA datasets [32]. Astrocyte-like cells showed the highest predicted signaling activity, primarily in an autocrine manner (124 interactions, strength 2.62), but also through paracrine signaling, sending 74 potential signals to oligodendrocyte-like cells (strength 2.53) and 74 signals to NPC-like cells (strength 1.88) (Fig. 7A). These analyses suggest that astrocyte-like cells may engage in a greater number of signaling interactions compared to other cell populations in the LGG275 line.

Among the identified signaling pathways (Fig. 7B), the NOTCH pathway was of particular interest given its reported roles in glioma cell quiescence and phenotype, as well as its role in astrocyte differentiation from NSC [6, 17]. In addition, NOTCH signaling has been associated with tumor grade and prognosis in gliomas, some studies highlight that NOTCH pathway inactivation is associated with advanced disease and shorter survival [72, 73]. Moreover, recent work in IDH-mutant oligodendroglioma suggests that NOTCH1 mutations are associated with reduced astrocytic differentiation and may limit the response to IDH inhibitors [74]. CellChat and RNA-sequencing analysis indicated that astrocyte-like cells preferentially expressed *NOTCH2* receptor as well as the ligand *JAG1*, while oligodendrocyte-like cells were enriched for *NOTCH1* receptor alongside the ligand *DLL1* (Fig. 7C, D). RNA-sequencing further showed that astrocyte-like cells display higher expression of downstream NOTCH targets (*HEY1, HEY2, HES5*) than oligodendrocyte-like cells, which were enriched in *DLL3* and *HES6,* two negative regulators of NOTCH signaling [75, 76]. To further assess NOTCH pathway activity in CD44⁺GLAST⁺ and CD44⁻GLAST⁻ cells, we performed GSEA on the RNA-sequencing data from these two populations using two in-house gene sets representing genes significantly up- or downregulated following DLL4 ligand-induced NOTCH activation in LGG275 (Table S4) [6]. These analyses indicated that CD44⁺GLAST⁺ cells are enriched for NOTCH-activated gene signatures compared to CD44⁻GLAST⁻ cells (Fig. 7E).

**Fig. 8:**
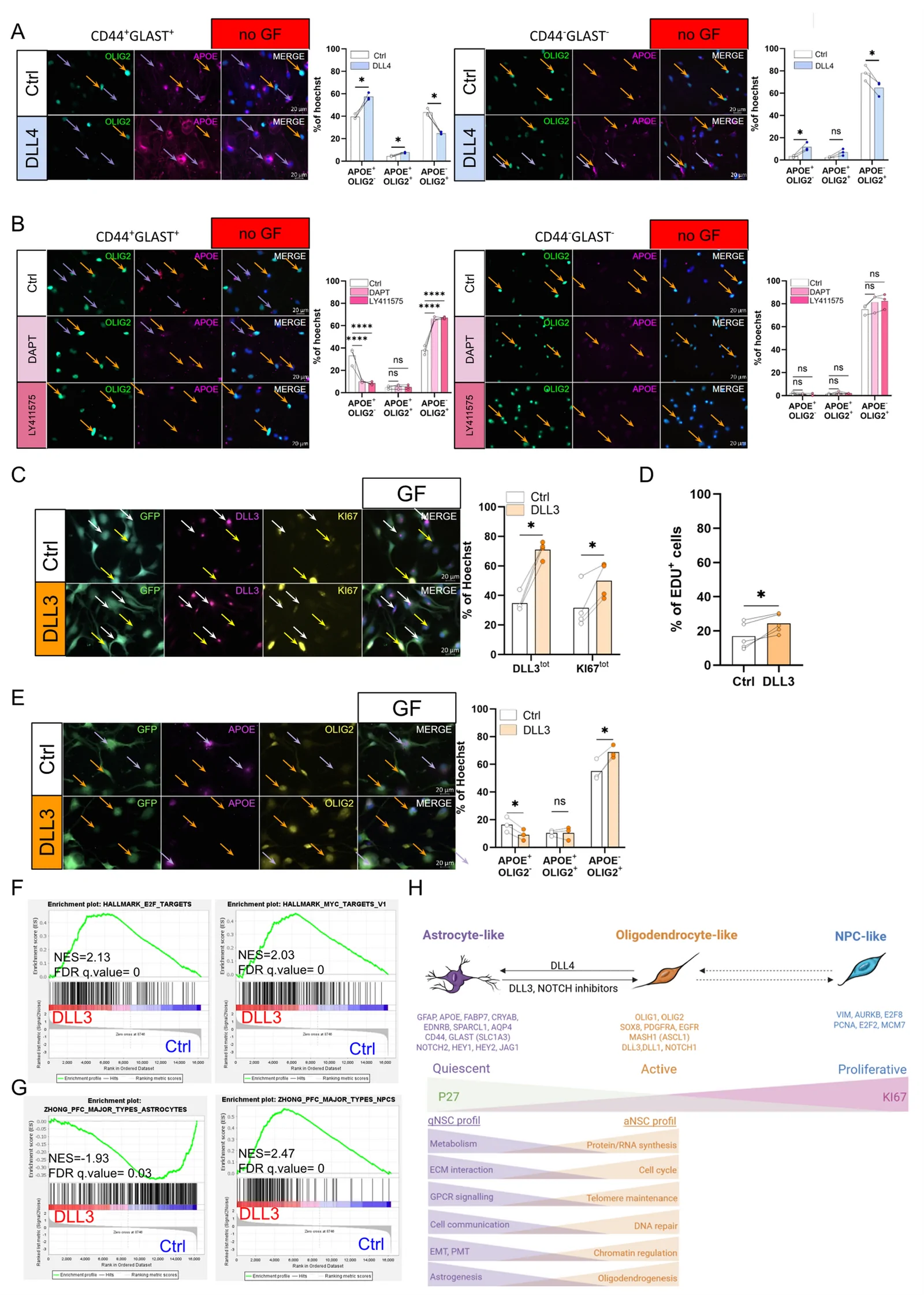
The NOTCH pathway is involved in the cellular plasticity of astrocyte-like and oligodendrocyte/NPC-like cells. **A**, **B** IF analysis of APOE and OLIG2 expression in the LGG275 line, identifying “astrocyte-like” APOE⁺OLIG2⁻ cells (purple arrows) and “oligodendrocyte/NPC-like” APOE⁻OLIG2⁺ cells (orange arrows) without GF, in CD44^+^GLAST^+^ and CD44^−^GLAST^−^ populations. Cells were treated with the NOTCH ligand DLL4 (**A**) and γ-secretase inhibitors (DAPT, LY411575) (**B**). **C** IF analysis of DLL3 and KI67 expression in the LGG275 line identifying DLL3^+^ (white arrows) and mitotic KI67^+^ (yellow) populations with GF, after overexpression of DLL3. **D** EDU incorporation analysis identifying proliferative EDU^+^ cells in DLL3 overexpressing cell vs Ctrl cells. bar plots show quantification as % of EDU⁺ cells (Bars = mean, dots = n = 3) (*p < 0.05, Student’t test) (**E**) IF analysis of APOE and OLIG2 expression in the LGG275 line, identifying “astrocyte-like” APOE⁺OLIG2⁻ cells (purple arrows) and “oligodendrocyte/NPC-like” APOE⁻OLIG2⁺ cells (orange arrows) with GF, after overexpression of DLL3. For A, B, C and E, bar plots show quantification as % of Hoechst⁺ cells (Bars = mean, dots = n = 3) (ns *p*> 0.05, *p < 0.05, and ****p< 0.0001, Dunnett’s multiple comparison test (**A**) or Student’s multiple test (**B**, **C**, **E**). **F**, **G** GSEA analyzes showing enrichment of proliferation-associated signatures (E2F and MYC targets) following DLL3 overexpression (**F**), and differential enrichment of astrocyte and NPC gene signatures (**G**) in control (Ctrl) and DLL3-overexpressing conditions, respectively (n = 3). NES = Normalized Enrichment Score; FDR = False Discovery Rate. **H** Schematic summary of the cellular states identified in the LGG275 astrocytoma cell line. Astrocyte-like cells correspond to a quiescent state that can transition to an active oligodendrocyte-like state and subsequently to a proliferative NPC-like state following DLL3 or NOTCH pathway inhibition. Conversely, activation of the NOTCH pathway promotes maintenance or re-entry into the quiescent astrocyte-like state. These three states display molecular and functional profiles resembling qNSC, aNSC, and proliferative neural stem cells (NSC), respectively

To test NOTCH-dependent plasticity, purified CD44⁺GLAST⁺ and CD44⁻GLAST⁻ cells were treated with DLL4 ligand for 6 days and APOE/OLIG2 expression was assessed by IF. DLL4 increased APOE⁺OLIG2⁺ astrocyte-like cells (~ 39 →  ~ 57%) and reduced APOE⁻OLIG2⁺ oligodendrocyte/NPC-like cells by about half, with similar effects in CD44⁻GLAST⁻ cells (Fig. 8A), consistent with promotion of an astrocyte-like state. Conversely, NOTCH inhibition (DAPT and LY411575) in purified CD44⁺GLAST⁺ cells decreased APOE⁺ cells and induced OLIG2 expression, while having minimal effects in purified CD44⁻GLAST⁻ cells (Fig. 8B), suggesting low baseline NOTCH activity in this population.

### DLL3 regulates proliferation and phenotype of LGG275 cells

Finally, given the association of NOTCH signaling with astrocyte-like and oligodendrocyte-like states in LGG275, we sought to identify potential modulators of this pathway. We focused on DLL3, a reported negative regulator of NOTCH activity [75]. In astrocytoma patients, analysis of the snRNA-seq dataset from Blanco-Carmona et al. [24] showed that DLL3 is predominantly expressed in oligodendrocyte-like, RE (ribosomal-enriched, OPC-related progenitor-like), and cycling tumor cell populations (Fig. S3H). Consistently, in LGG275, scRNA-sequencing indicated that DLL3⁺ cells were mainly oligodendrocyte-like, a distribution that persisted upon growth factor withdrawal (Fig. S10A). Bulk RNA-sequencing and proteomic analyses further supported the preferential expression of DLL3 in oligodendrocyte-like cells (Fig. 5C, D**)**. IF shows that DLL3 is expressed in a subset (30–40%) of cells, with a Golgi-associated localization consistent with previous reports [77] (Fig. 8C and S10B, C). Co-staining with OLIG2 indicated that DLL3⁺ cells are mainly OLIG2⁺, both in the presence and absence of GF (Fig. S10B). In proliferating cultures, DLL3⁺ cells displayed mixed KI67 and P27 expression profiles (Fig. S10B,C), suggesting that this population may occupy an intermediate cell-cycle state.

To explore the potential role of DLL3 in LGG275 cell behavior, we overexpressed DLL3 using a lentiviral vector, which approximately doubled the proportion of DLL3⁺ cells (Fig. 8C). This was associated with an increase in KI67⁺ and EDU⁺ cells, consistent with enhanced proliferative activity (Fig. 8C, D). DLL3 overexpression was also accompanied by a decrease in APOE⁺OLIG2⁻ cells and an increase in APOE⁻OLIG2⁺ cells (Fig. 8E). Consistently, GSEA of RNA-seq data from DLL3-overexpressing cells revealed enrichment of NPC-related and proliferation-associated signatures, alongside a reduction in astrocyte-associated programs (Fig. 8F, G; Fig. S10D–G; Tables S15 and S16). Similar trends were observed in LGG336, where DLL3 overexpression was associated with increased proliferation marker KI67, EDU^+^ cells, and reduced GFAP expression (Fig. S11A–C). Together, these findings are consistent with a role for DLL3 in modulating proliferation and lineage plasticity.

## Discussion

In this study, we established and comprehensively characterized four newly generated cell lines derived from IDH-mutant astrocytomas. Multi-omics profiling and functional studies, particularly of the slow-growing LGG275 line, yielded several key insights into the biology of IDH-mutant astrocytomas, summarized in Fig. 8H.

First, glioma cell lines established under serum-free conditions are typically maintained in non-adherent cultures, allowing the formation of neurospheres [37, 38]. However, we observed that for two lines (LGG275 and LGG349), this culture mode was not favorable for cell expansion. When grown on laminin, these cells displayed improved growth, suggesting that integrin-mediated signaling may be involved and could confer a proliferative advantage, as previously reported for non-tumoral human neural stem cells [78]. In contrast, the LGG336 line showed no major difference between the two culture modes, whereas LGG85 grew preferentially under non-adherent conditions. These findings highlight heterogeneity in adhesion-dependent growth requirements among the lines, potentially reflecting differences in the signaling pathways that are active in each of them. Additional heterogeneity was observed in the stability of the IDH1 mutation. While three lines (LGG85, LGG336, and LGG275) retained the mutation beyond 10–15 passages, it was lost in LGG349 despite its presence in the original tumor. This observation may reflect selection during culture of a pre-existing IDH1-wild-type subclone present in the LGG349 tumor, or loss of the mutation over passages, as previously reported [42]. Notably, LGG85, which is derived from a high-grade tumor, retained the mutation, indicating that loss of IDH1 is not strictly associated with tumor grade. 

Second, an important observation arises from the comparison between lines derived from low-grade and high-grade tumors, which respectively fail to form or successfully form tumors in vivo, thereby modeling the behavior of the original patient’s tumors. The LGG85 line exhibits a high number of mutations, likely because of temozolomide treatment [41], whereas LGG349 shows strong expression of c-MET and PDGFRA, both known oncogenic drivers of tumor progression [5]. Moreover, LGG349 expresses developmental genes (such as HOX genes) that are normally silent in the adult brain [79]. These findings suggest that multiple distinct mechanisms can underlie tumor progression, and that these two lines will be valuable tools to dissect the molecular pathways driving malignancy. It is also important to note that although the LGG85 and LGG349 lines display high-grade characteristics, they grow more slowly than classic GB cells, underscoring the remarkable heterogeneity among high-grade gliomas, which likely results from distinct mechanisms of transformation. A salient feature distinguishing the lines derived from low- and high-grade gliomas concerns the persistence of NPC-like cells upon GF withdrawal. In both high-grade-derived lines, the proportion of NPC-like cells remains largely unchanged in the absence of GF, whereas it is markedly reduced in LGG275 and LGG336. The mechanisms sustaining NPC-like cell persistence under growth factor deprivation remain to be fully elucidated but may be related to the increased mutational burden observed in these lines [7].

Third, scRNA-sequencing and IF analyses indicated the coexistence of multiple transcriptional states within each line, corresponding to astrocyte-like, oligodendrocyte-like, and NPC-like phenotypes similar to those identified in patient-derived gliomas [6–8]. The boundaries between these states were particularly sharp in the LGG275 line but became blurred in the high-grade lines, suggesting that tumor progression may be associated with reduced cell state specificity. This loss may stem from extensive epigenetic remodeling or disruptions in the underlying gene regulatory networks caused by patient-specific mutations. Compared to grade 2/3 IDH-mutant astrocytomas, in which proliferative NPC-like cells typically represent only 2–5% of the tumor, the relative enrichment of NPC-like cells in our cultures may reflect the high levels of exogenous growth factors as well as the absence of a tumor microenvironment, which plays a key role in regulating cellular states [80, 81], for instance through endothelial-derived signals such as endothelins [82]. In addition, adaptation to in vitro culture conditions, potentially accompanied by the emergence of additional mutations, may also contribute to this shift in cellular composition. However, upon growth factor withdrawal in the LGG275 cell line, cycling cells were reduced to ~ 4%, a proportion comparable to that observed in patient tumors.

Fourth, using LGG275 as a reference model, we identified two surface markers, CD44 and GLAST that can be used to isolate astrocyte-like cells for functional and molecular analyses. These experiments revealed that astrocyte-like cells tend to reside in a more quiescent state but retain the capacity to exit quiescence and transition toward an oligodendrocyte-like phenotype. Morphologically, astrocyte-like and oligodendrocyte-like populations are also clearly distinct: astrocyte-like cells are larger and more flattened, whereas oligodendrocyte-like cells display a bipolar, elongated morphology. Recent study [83] has demonstrated that oligodendrocyte-like tumor cells make a predominant contribution to glioma infiltration. Their bipolar morphology observed here may therefore be consistent with features associated with migratory and invasive behavior within the brain parenchyma., Transcriptomic and proteomic comparisons between astrocyte-like and oligodendrocyte-like populations further revealed similarities with SVZ qNSC and aNSC states, respectively. These similarities extend, for instance, to metabolic programs: based on bioinformatic analysis of the proteomic data, astrocyte-like cells showed enrichment for metabolic programs associated with fatty acid oxidation, a feature linked to quiescent stem cells [66]. In the adult SVZ, the establishment and maintenance of qNSC are tightly controlled by the NOTCH signaling pathway [84–86]. Consistent with this, our analyses suggest that NOTCH signaling contributes to the balance between astrocyte-like and oligodendrocyte-like populations in LGG275 cells. Among NOTCH-related molecules, we identified DLL3, an inhibitory ligand, as a candidate modulator [77]. DLL3 has been associated with favorable prognosis, with expression inversely correlated with tumor grade [87, 88]. It is also targeted by emerging therapies aimed at inducing tumor cell death [89], underscoring its potential relevance in cellular plasticity. In LGG275, DLL3 is predominantly expressed in oligodendrocyte-like cells at both the RNA and protein levels, as supported by scRNA-sequencing, IF, and proteomic analyses, consistent with its expression in astrocytoma single cell seq dataset (Fig. S3H) [24]. Functionally, DLL3 overexpression was associated with reduced expression of some astrocytic genes and increased expression of proliferation-associated genes, consistent with a potential role for DLL3 in maintaining a more activated, proliferative state of oligodendrocyte-like cells, possibly through modulation of NOTCH activity [90]. Collectively, these findings position DLL3 as both a potential marker and a putative regulator of oligodendrocyte-like glioma cells, supporting its involvement in shaping cell-state balance and plasticity in IDH-mutant astrocytomas.

### Limitations

Our study has several limitations. First, the absence of matched germline and primary tumor DNA precluded comprehensive mutational analyses, preventing us from distinguishing true somatic alterations from potential culture-acquired changes. Second, although the IDH1 mutation was stably maintained in our cell lines for more than 10 passages, its long-term stability remains uncertain. It will be important to determine whether extended culture leads to loss of the mutation and whether this is associated with reduced cellular heterogeneity or a shift toward a more homogeneous state. Third, while we identified distinct astrocyte-like, oligodendrocyte-like, and NPC-like states in vitro, we did not perform single-cell RNA sequencing on xenografted tumors. Such analyses will be necessary to assess whether these cellular states are preserved in vivo. Fourth, although NOTCH signaling and DLL3 modulate astrocyte-like and oligodendrocyte-like states in IDH1-mutant lines, this could not be evaluated in the high grade LGG85 and LGG349 lines, limiting generalizability. Fifth, we did not derive or analyze IDH-wild-type low-grade astrocytoma cell lines, nor did we engineer LGG275 to ablate the *IDH1* mutation. Including such models would be important to determine whether the transcriptional states described here, and their regulation by NOTCH and DLL3, are specific to IDH-mutant tumors. Finally, we were not able to derive longitudinal cell line models from the same patient at different stages of disease progression, which would have enabled us to directly assess whether NOTCH signaling becomes attenuated during progression, as recently suggested [73]. In addition, a systematic evaluation of NOTCH pathway activity in the original patient tumors used to derive these cell lines would have strengthened the integration of our findings with disease progression and enhanced their clinical relevance.

## Conclusion

We established and extensively characterized four cell lines derived from IDH1-mutant astrocytomas that recapitulate several molecular and phenotypic features of patient tumors, including partial or complete activation of ALT, canonical mutations (*IDH1, TP53, ATRX, MET*), and grade-consistent growth behaviors in vitro and in vivo. These cell lines, supported by detailed multi-omics annotations, provide a valuable resource for glioma research. They may enable the investigation of key pathogenic mechanisms, including ALT biology, progression to high-grade disease, and responses to emerging therapies, and offer a relevant platform to dissect the molecular mechanisms underlying distinct tumor cell states. Among them, LGG275 may represent a particularly relevant model for studying slow-growing IDH-mutant astrocytomas.

## Supplementary Information

Below is the link to the electronic supplementary material.

Supplementary Material 1.

Supplementary Material 2.

Supplementary Material 3.

## Data Availability

The datasets supporting the conclusions of this article are included within the article and Supplemental materials. The data presented in this study are available in this article and Supplemental materials. Bulk and single cell RNA-seq datasets have been deposited in the NCBI Gene Expression Omnibus (GEO) under the following accession numbers: Single-cell RNA-sequencing datasets can be accessed at GSE263796. RNA-sequencing of the 4 astrocytoma lines can be accessed at GSE308063, RNA sequencing of the CD44 + GLAST + and CD44-GLAST- subpopulations can be accessed at GSE305451, RNA-sequencing of mitotic (P27-) and quiescent (P27 +) cells can be accessed at GSE306414, RNA-sequencing of the effect of DLL3 overexpression can be accessed at GSE308910. Whole exome-sequencing datasets can be accessed in the SRA database at PRJNA1345048. Bulk and single cell RNA-seq datasets have been deposited in the NCBI Gene Expression Omnibus (GEO) under the following accession numbers: Single-cell RNA-sequencing datasets can be accessed at GSE263796. RNA-sequencing of the 4 astrocytoma lines can be accessed at GSE308063, RNA sequencing of the CD44^+^GLAST^+^ and CD44^−^GLAST^−^ subpopulations can be accessed at GSE305451, RNA-sequencing of mitotic (P27^−^) and quiescent (P27^+^) cells can be accessed at GSE306414, RNA-sequencing of the effect of DLL3 overexpression can be accessed at GSE308910. Whole exome-sequencing datasets can be accessed in the SRA database at PRJNA1345048.
