# Supplementary material for: Multi-omics characterization of IDH-mutant astrocytoma-derived cell lines reveals NOTCH-regulated plastic quiescent astrocyte-like state and insights into progression
Source: Acta Neuropathol Commun. 2026 Jul 20;14:221. doi: 10.1186/s40478-026-02316-9 (PMC13693255; doi:10.1186/s40478-026-02316-9)
Supplement: Supplementary file 1 [file 40478_2026_2316_MOESM1_ESM.pdf]

Figure 1

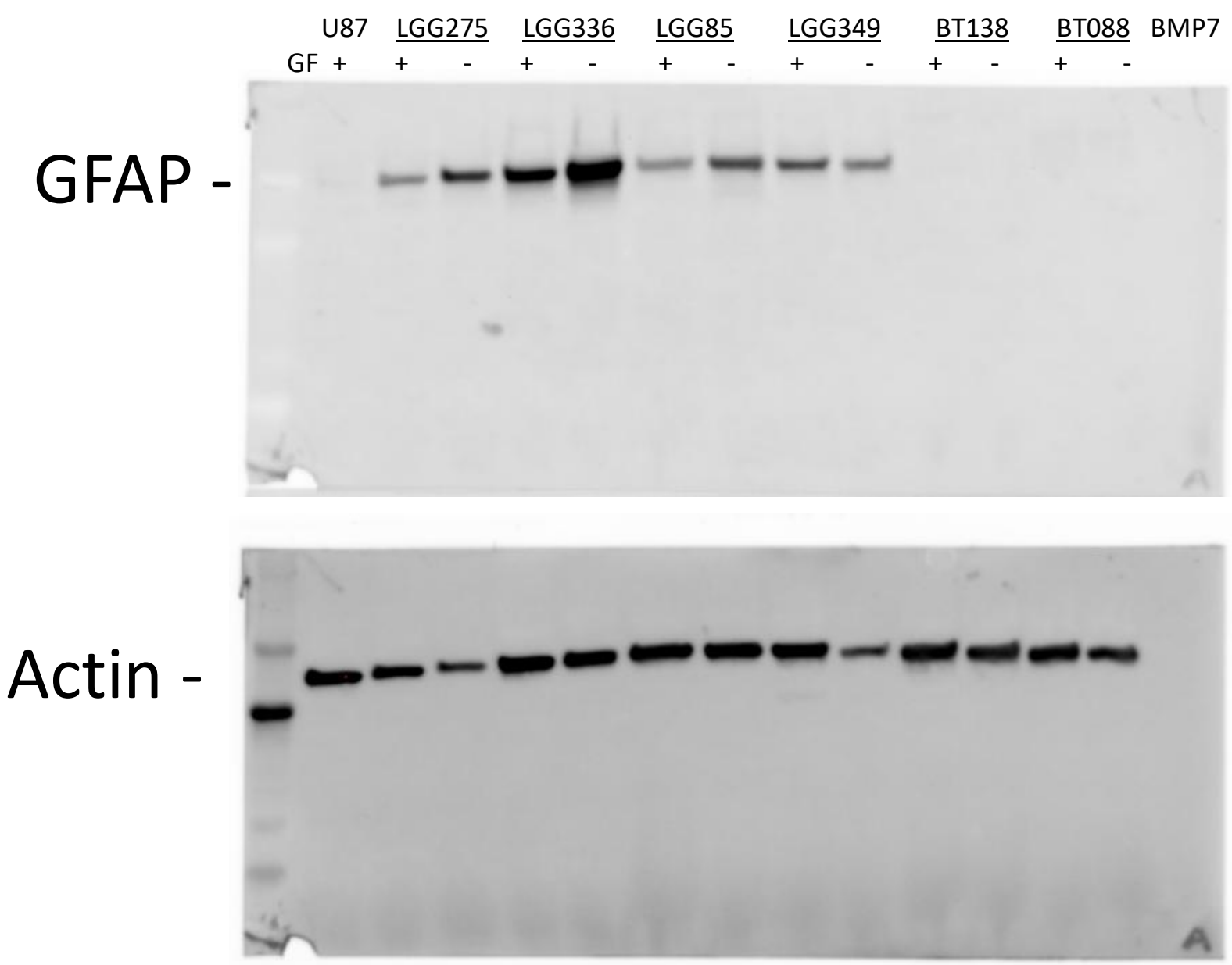

Figure 2

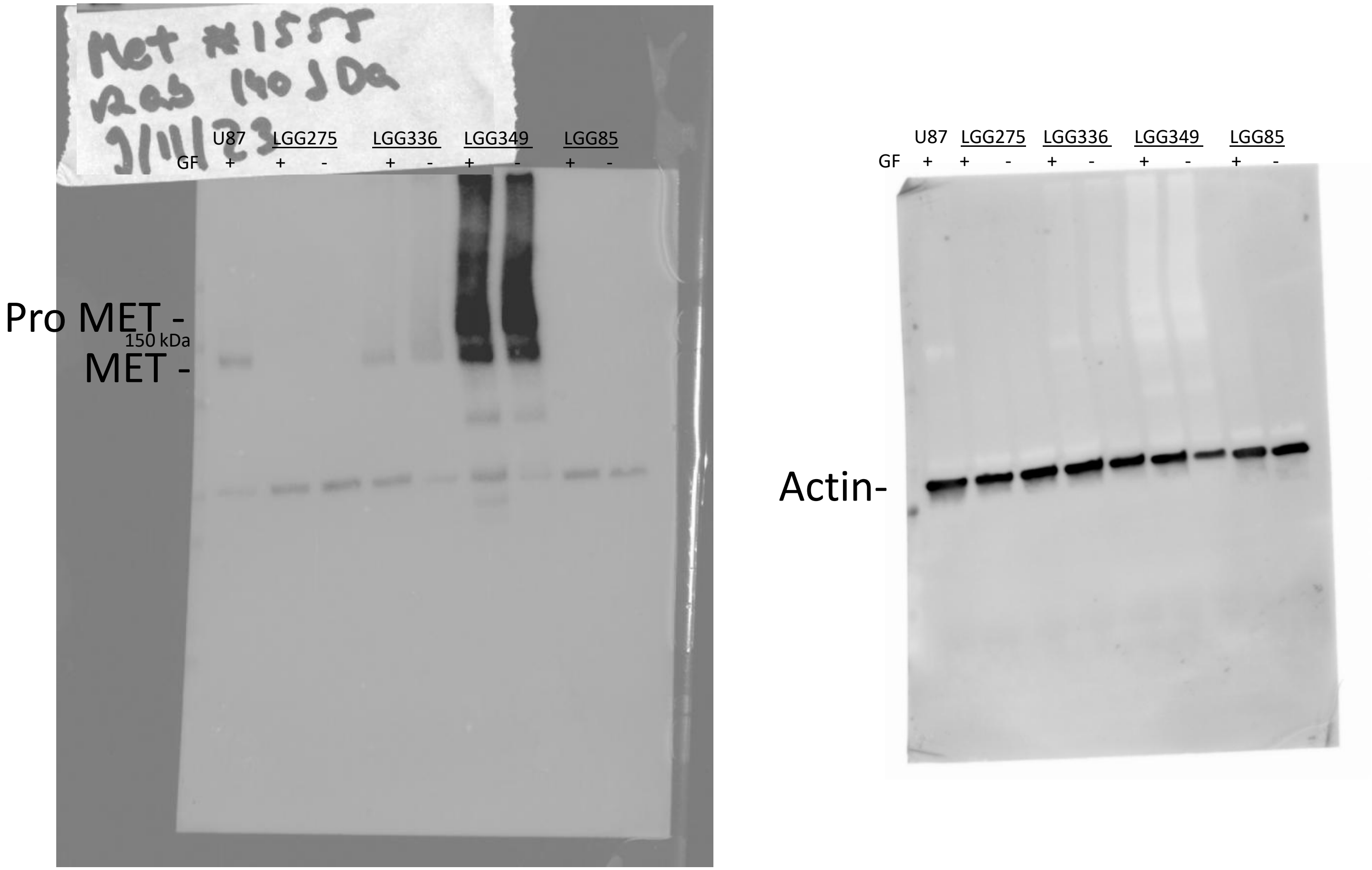

Figure 2

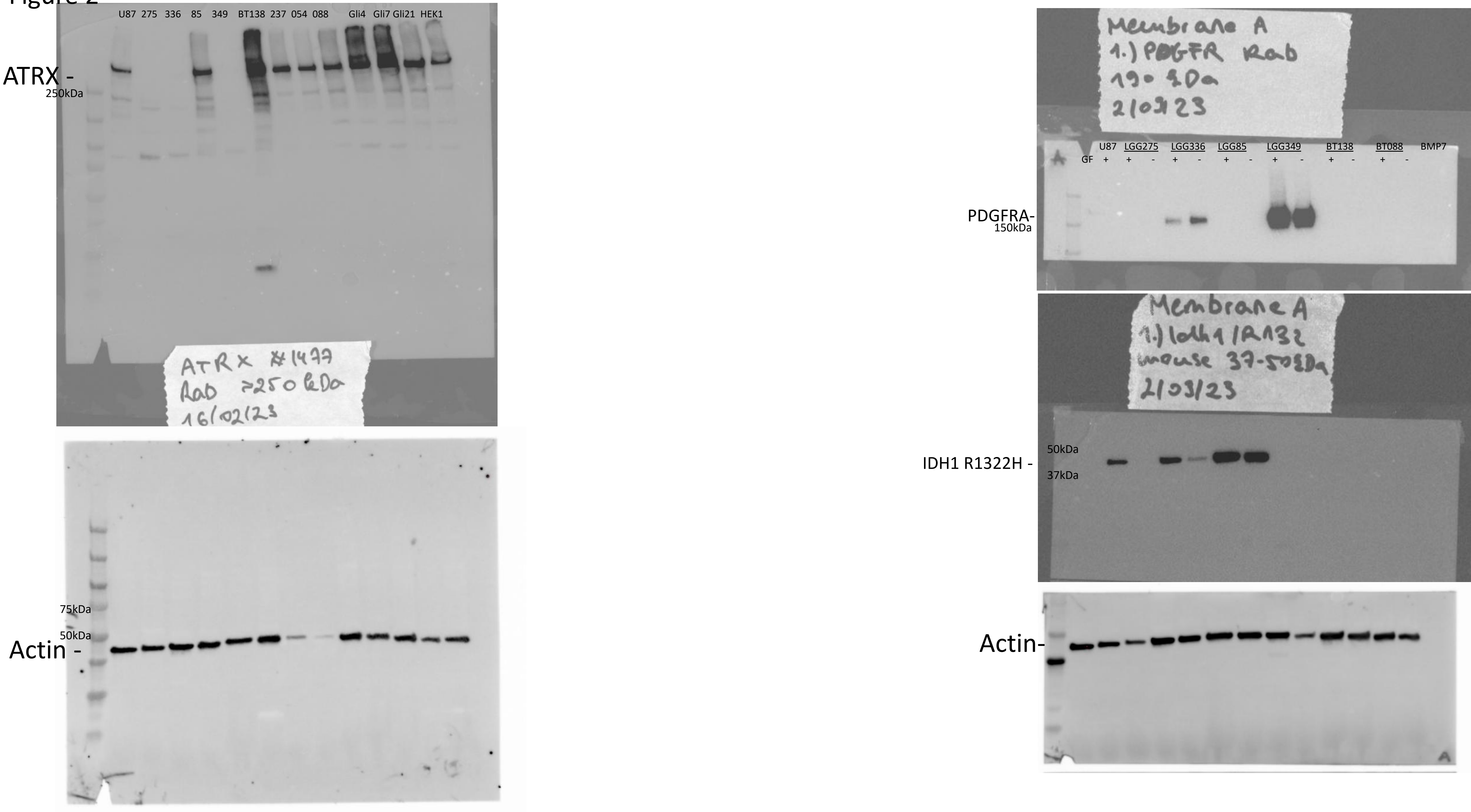

U87 275 336 85 349 BT138 237 054 088 Gli4 Gli7 Gli21 HEK1
ATRX -
250kDa
ATRX #1477
Rab >250 kDa
16/02/23
75kDa
50kDa
Actin -
Membrane A
1.) PDGFR Rab
190 kDa
2/03/23
U87 LGG275 LGG336 LGG85 LGG349 BT138 BT088 BMP7
GF + + - + - + - + - + - + -
PDGFRA-
150kDa
Membrane A
1.) Idh1/R132
mouse 37-50kDa
2/03/23
50kDa
IDH1 R1322H -
37kDa
Actin-
