# Supplementary material for: Multi-omics characterization of IDH-mutant astrocytoma-derived cell lines reveals NOTCH-regulated plastic quiescent astrocyte-like state and insights into progression
Source: Acta Neuropathol Commun. 2026 Jul 20;14:221. doi: 10.1186/s40478-026-02316-9 (PMC13693255; doi:10.1186/s40478-026-02316-9)
Supplement: Supplementary file 2 [file 40478_2026_2316_MOESM3_ESM.docx]

**Supplemental Methods and Materials**

**Table of Contents**

**Supplemental Methods	Page 2**

**Supplemental Figures	Page 7**

**Supplemental Figure Legends	Page 19**

**Supplemental Methods**

**RNA Extraction, qPCR, RNA seq**

RNA was extracted using RNeasy Mini or Micro kits according to the manufacturer’s instructions (Qiagen). For qPCR, cDNA was synthesized from 200 ng RNA using random primers and GoScript™ reverse transcriptase. qPCR was performed in triplicate with 2 ng cDNA using the KAPA SYBR® FAST kit on a LightCycler 480 (Roche). Primers are listed in **Table.S3**. Gene expression was calculated using the 2^−ΔΔCt^ method, with β-actin (ACTB) as the housekeeping gene. RNA sequencing was performed by BGI (DNBSEQ platform). Libraries were prepared using a dual-mode mRNA kit, including poly(A) selection, fragmentation, cDNA synthesis, adapter ligation, and PCR amplification. Sequencing was carried out on a DNBSEQ-T7 platform (PE150, ~20M paired reads/sample). Raw data were filtered using SOAPnuke (v1.5.2), and differential expression analysis was performed with DESeq2. Data visualization was generated using SRPlot (<https://www.bioinformatics.com.cn>).

**Whole Exome Sequencing**

Genomic DNA was extracted from the four astrocytoma cell lines (between 10-13 passages) using a DNA Purification kit (Promega) and sequenced by BGI on the DNBSEQ platform using DNBSEQ-G400. BGI also performed sample preparation and bioinformatic analyses. Samples were subjected to quality control according to sample type and products requirements. Genomic DNA was randomly fragmented, selected to a certain average size and subjected to end-repair and then was 3 adenylated. Adaptors were ligated to the ends of these 3 adenylated fragments. Product was amplified by PCR, then purified. Hybridization were performed. Single-stranded PCR products are produced via denaturation, then cyclized. Uncyclized linear DNA molecules are digested. Single-stranded circle DNA molecules are replicated via rolling cycle amplification, and a DNA nanoball (DNB) which contain multiple copies of DNA is generated. Sufficient quality DNBs are then loaded into patterned nanoarrays using high-intensity DNA nanochip technique and sequenced through combinatorial Probe-Anchor Synthesis (cPAS). Raw data were cleaned using SOAPnuke filter. Clean datas were then aligned using the human reference genome GRCh38-p13, Burrows-Wheeler Aligner (BWA V0.7.17 - <http://bio-bwa.sourceforge.net/>) and BWA-MEM method. Duplicate reads were removed using GATK MarkDuplicates tool (v4.1.4.1, <https://gatk.broadinstitute.org/hc/en-us/articles/360037225972-MarkDuplicates>). Base quality values have been improved using GATK BaseRecalibrator (v4.1.4.1, <https://gatk.broadinstitute.org/hc/en-us/articles/360037593511-BaseRecalibrator>) and GATK ApplyBQSR (v4.1.4.1, <http://bio-bwa.sourceforge.net/>) softwares. Known mutations and single nucleotide polymorphisms (SNPs) in each cell line were identified using the VarDecrypt software.

**Western Blot Analysis**

Cells were lysed in RIPA buffer with protease and phosphatase inhibitors. Protein concentration was determined by BCA assay. Proteins were separated by SDS-PAGE, transferred to PVDF membranes, and incubated with primary antibodies (**Table.S3**) overnight, followed by HRP-conjugated secondary antibodies. Signals were detected using ECL and imaged with a ChemiDoc system.

**Measurement of Intracellular 2-HG Quantities in Cell Lines**

Intracellular 2-hydroxyglutarate (2-HG) levels were measured using a 2-HG fluorimetric assay kit, following the manufacturer’s instructions. A total of 2 million cells were used per sample. For analysis, the values from blank wells were subtracted from both standards and samples.

**Telomere-FISH**

Telomere-fluorescence in situ hybridization (Telo-FISH) was performed on metaphase spreads. Cells were cultured flasks treated with Karyomax, harvested using trypsin, and resuspended in hypotonic solution (0.6% sodium citrate). Cells were then fixed with ethanol/acetic acid (3V:1V). Metaphase chromosome preparations were obtained by spreading cells on SuperFrost microscope slides using a cytogenetic drying system (Thermotron AdGenix). After drying, Telo-FISH staining has been performed as previously described [21] . Chromosomes were counterstained with DAPI. Zeiss Axioplan2 microscope with Metafer imaging software was used to image metaphases. For each cell line, 80 to 208 metaphases were analyzed.

**Telomeric Repeat Amplification Protocol (TRAP)**

Telomerase enzymatic activity was determined using the TRAPeze Telomerase detection kit according to the manufacturer's instructions. Protein concentrations were determined using the Bradford method. Telomerase activity was assessed and TRAP products were separated on a 12.5% non-denaturing polyacrylamide gel electrophoresis (PAGE) in 0.5X TBE buffer. Gels were stained with ethidium bromide and PCR amplification products were visualized under UV light exposure.

**Colocalization of PML/Telomeres and Detection of ALT-Associated PML Bodies (APBs)**

Cells were fixed with 4% PFA, and permeabilized with PBS, 0.5% Triton. Blocking was performed in PBS, 7.5% fetal calf serum, 7.5% goat serum. Blocked cells were incubated overnight an α-PML antibody, and 1h a secondary antibody. After incubation; slides were fixed in 4% formaldehyde, dehydrated in a graded ethanol solution (50%, 80%, and 100%) and incubated with a Cy3-telomeric PNA probe (CCCTAA)_3_ diluted in a hybridization solution (70% formamide, 10 mM Tris-HCl pH7.2, 1% BSA) followed by hybridization. Cells were counterstained with DAPI. Slides were mounted using Fluoromount. Images were captured using a Nikon A1 confocal microscope (objective x 60) and 29 to 168 cells were analyzed per astrocytoma.

**C-circle Assay**

Amplification and detection of c-circles were performed as described by Henson [22] , with minor modifications. DNA extraction was performed with QIAamp DNA Blood Mini Kit, then amplified with Φ29 polymerase. Telomeric qPCR was used to determine CCA levels by measuring the increase in total telomeric DNA produced during the assay and normalized to the total DNA amount determined by qPCR for the 36B4 gene.

**Cell Proliferation Analysis by Dye Dilution**

LGG275 cells were labeled with CellTrace™ according to the manufacturer’s instructions, then seeded at 25,000 cells/cm² and cultured ± GF for 20 days. Cells were dissociated and CellTrace™ dilution was analyzed by flow cytometry (MACSQuant, Miltenyi) using FlowJo.

**Cell Cycle Analysis**

Cells were seeded at 30,000 cells/cm² and cultured for 20 days ± GF, or 10 days - GF followed by 10 days +GF. Cells were dissociated, fixed in 70% ethanol, treated with RNAse, and stained with propidium iodide. Cell cycle distribution was analyzed by flow cytometry (MACSQuant, Miltenyi) using FlowJo.

**Gene Set Enrichment Analysis (GSEA)**

The GSEA analysis software (Version 4.3.3) was used to perform a comparative analysis of differentially expressed genes and molecular profiles between different conditions after RNA sequencing. The compared gene lists are the “normalized read count”. The gene ranking method is the “Signal2Noise” method for groups consisting of triplicates and “Diff of class” for groups composed of duplicates. The collections used for comparison in this paper are the “Curated c2” collections (c2.all. v2024.1.Hs.symbols.gmt), “Cell types c8” (c8.all. v2024.1.Hs.symbols.gmt), “Gene Ontology c5” (c5.all. v2024.1.Hs.symbols.gmt) and “Computational c4” (c4.all. b2024.Hs.symbols.gmt). The “quiescent NSC1” (qNSC1), qNSC2 and “active NSC” (aNSC) gene lists were established from Kalamakis et al [34] . The p.value was calculated using the Benjamini-Hochberg method. NOTCH_UP and NOTCH_DOWN genelist were constructed by treated cells with DLL4, an activator of NOTCH, then isolate significant up (NOTCH_UP) and down (NOTCH_DOWN) regulated genes following NOTCH activation (**Table.S4**).

**Cytoscape Mapping**

GSEA results (GO c5 collection) were visualized using Cytoscape (v3.3). Terms were filtered (p < 0.05, FDR q < 0.25, overlap coefficient < 0.5), highlighting shared genes across enriched pathways.

**Lentivirus Infections**

LGG275 cells were transduced with luciferase-GFP or DLL3-GFP constructs. For the P27 reporter, cells were infected with control mVenus-BFP2 or P27-mVenus-BFP2 (MOI 7)[36]. Transduced cells were expanded and sorted based on GFP or BFP2 expression using a FACSAria (**Table.S5**).

**Treatments of Subpopulations with DLL4 and γ-Secretase Inhibitors**

For DLL4 stimulation, PDL-coated coverslips were incubated overnight at 4°C with DLL4 or 0.1% BSA (control) prior to cell seeding. For γ-secretase inhibition, cells were plated on PDL/laminin-coated coverslips, cultured for 6 days without GF, and treated every 24 h with DMSO, DAPT, or LY411575.

**Supplemental Figures**

**Figure S1 (related to Figure 1 and 2): In vivo cell dissemination and altered gene expression in four IDH-mutant astrocytoma cell lines** (A) Grafted cell spread area in the mouse brain 6 months after intracranial injection of LGG275 and LGG85 cells. For each mouse, representative sections were imaged and used to quantify the invaded area (detection of tumoral cells with human-specific Nestin antibody. Bars represent mean values and dots individual mice (n = 3). *P < 0.05 (Student’s t-test). (B) Variant allele frequency (VAF) of the IDH1 c.395G>A (R132H) mutation in the corresponding patient tumors and derived cell lines (passages 10–13). (C–D) mRNA expression levels of MET (C) and PDGFRA (D), expressed as TPM (transcripts per million), in LGG275, LGG336, LGG85, and LGG349 (n = 1). (E) Immunofluorescence analysis of MET expression in LGG349 tumor tissue and corresponding cell line. Scale bars: 50 µm (tissue) and 20 µm (cell line).

**Figure S2 (related to Figure 3): scRNA sequencing and RNA sequencing analysis of 4 astrocytoma cell lines** (A) GSEA analysis showing enrichment of astrocyte, oligodendrocyte and NPC signatures in astrocyte-like, oligodendrocyte-like and NPC-like subpopulation, respectively, in LGG275 cell lines, with or without (growth factors. q.value was calculated using Benjamini-Hochberg methods. NES = Normalized Enrichment Score, FDR = False Discovery Rate (B) Stacked bar plots showing, for each glioma cell line (LGG275, 336, 85, 349), the proportion of reference signature genes (light blue; astrocyte-like, NPC-like, and oligodendrocyte-like gene sets) that are recovered among significant differentially expressed marker genes (dark blue). The y-axis indicates the number of genes in each list (total reference set in light blue, with the overlapping significant DEGs highlighted in dark blue). Reference signature gene lists were curated in-house as described in the Materials and Methods. Percentages indicate the fraction of each reference gene set overlapping with the significant DEG. (C-E) Dot Plot of astrocyte, oligodendrocyte and proliferative markers expression in each subpopulation of LGG336 (C) LGG85 (D) and (E) LGG349 with growth factors. (F)  mRNA expression (TPM) of proliferation-associated genes in four astrocytoma cell lines cultured with growth factors (GF) or without growth factors (noGF). Bars show the mean across cell lines; dots represent individual cell lines (LGG275, LGG336, LGG349, LGG85; *n* = 4). Lines connect matched GF and noGF values for each cell line (G) Gene set enrichment analysis (GSEA) of RNA-seq data from four astrocytoma cell lines cultured with growth factors GF versus without growth factors (noGF) shows reduced enrichment of proliferation-associated programs upon GF withdrawal (HALLMARK_E2F_TARGETS and HALLMARK_MYC_TARGETS_V1). NES, normalized enrichment score; FDR, false discovery rate

**Figure S3 (related to Figure 3): Cross-validation of cell states in patient-derived snRNA-seq and scRNA-seq datasets of IDH-mutant astrocytomas.** (A–B, top) UMAP visualization and cell state proportions derived from a published single-nucleus RNA-sequencing (snRNA-seq) dataset of six grade 2/3 astrocytomas [23]. Cell state annotation was performed using either (A) our in-house cell type signatures or (B) previously defined Blanco-Carmona NMF-based signatures [23]. (B, bottom) Z-score heatmap showing the similarity between Blanco-Carmona signatures and LGG275 (no GF) cell type signatures (top 50 differentially expressed genes per state); positive values indicate higher concordance. (C) Dot plot from the Blanco-Carmona snRNA-seq dataset annotated using our gene signatures, showing the expression of the top five differentially expressed genes (DEGs) for each cell state, together with canonical markers of astrocytes, oligodendrocytes, and neural progenitor cells (NPC). (D) UMAP visualization and cell state proportions derived from a published scRNA-seq dataset of untreated grade 2/3 astrocytoma patients [7]. (E) Dot plot from the Venteicher et al. scRNA-seq dataset [7], displayed as in (C), showing the expression of the top five DEGs per cell state along with canonical astrocyte, oligodendrocyte, and NPC markers. (F) UMAP visualization and cell state proportions derived from an independent scRNA-seq dataset of grade 2/3 astrocytomas [24]. (G) Dot plot from the Chen et al. scRNA-seq dataset [24], displayed as in (C), showing the expression of the top five DEGs per cell state together with canonical lineage markers. (H) Violin plots of DLL3 expression (log2-transformed) across cell states in the Blanco-Carmona et al. astrocytoma dataset.
DLL3 expression levels are shown in astrocyte-like, cycling-like, gradient, OPC-like, and ribosome-enriched (RE) populations. Each violin represents the distribution of single-cell expression values, with width indicating cell density, highlighting differential expression across cell states.

**Figure S4 (related to Figure 3):   Further confirmation of distinct cellular states in cell lines**

(A) IF analysis of astrocytic markers (GFAP, FABP7) and oligodendrocyte/NPC-associated markers (MASH1, OLIG1) in the LGG275 cell line cultured with (GF) or without growth factors (noGF). Representative images (scale bar, 20 µm) and quantification of marker-defined subpopulations are shown (right). Cell counts were performed on 500 cells per condition (*n* = 1). (B) Flow cytometry analysis of CD44 and OLIG1 expression in the four astrocytoma cell lines cultured without growth factors (noGF), identifying astrocyte-like (CD44⁺OLIG1⁻; purple) and oligodendrocyte/NPC-like (CD44⁻OLIG1⁺; orange) populations (representative density plots shown). Barplot shows quantification as % of cells (Bars = mean, dots = n = 3).

**Figure S5 (related to Figure 4): Identification of a subpopulation of quiescent cells within LGG275 cell line**

(A) Cell cycle analysis by flow cytometry using propidium iodide in LGG275 cell line after 20 days with (GF) or without (no GF) GF or 10 days without GF following by 10 days with GF (no GF + GF). Right Panel: Barplot shows quantification as % of cells (bars = mean, dots = n = 3) (ns = non-significant, * p<0, 05, **<0,01, multiple comparison Turkey test) (B) GSEA analysis of RNA sequencing comparing mVenus/P27^+^ cells versus mVenus/P27^-^ cells shows a enrichment of cell cycle gene signatures in mVenus/P27^-^ cells in LGG275 line. q.value was calculated using Benjamini-Hochberg method. NES = Normalized Enrichment Score, FDR = False Discovery Rate. (C) Flow-cytometry analysis of mVenus–P27 fusion protein expression at 0, 4, 7, 11, and 15 days in sorted mVenus/P27⁻ and mVenus/P27⁺ populations. Representative density plots (FSC vs mVenus/P27) are shown. Right, quantification of mVenus/P27-negative, -low, and -high fractions as % of total cells. (bars = mean ± S.E.M, n=3).

**Figure S6 (related to Figure 4): Link between cellular state and quiescence in astrocytoma cell lines**

(A) IF analysis of APOE, OLIG2 and KI67 expression in LGG275 line, identifying KI67 preferential expression in oligodendrocyte/NPC-like (APOE^-^OLIG2^+^) rather than in astrocyte-like cells (APOE^+^OLIG2^-^) with and without GF, scale bar = 50µm. Right panels: Barplot shows quantification as % of Hoechst^+^ cells (bars = mean, dots = n = 3) (* p<0, 05, t-test) (B) left corner: UMAP colored by ccAFv2 cell-cycle state classifier, including Neural G0. Right, dot plot showing the association between phenotypic states (astrocyte-like, NPC-like, oligodendrocyte-like, unknown) and ccAFv2 cell-cycle classes (Pearson residual) in LGG336, LGG85 and LGG349 lines. Significance was assessed by a χ² test (p < 0.01) (C) IF staining of GFAP and KI67 in LGG85, LGG336, and LGG349 cells cultured with growth factors (GF), illustrating largely non-overlapping astrocyte-like (GFAP⁺; yellow arrows) and mitotic (KI67⁺; white arrows) populations. Representative images are shown; scale bar, 20 µm. Bottom: quantification of GFAP⁺KI67⁻, GFAP⁻KI67⁺, and GFAP⁻KI67⁻ fractions expressed as % of Hoechst⁺ nuclei (n=1, count = 500 cells).

**Figure S7 (related to Figure 5): CD44/GLAST-based cell sorting enables efficient isolation of astrocyte-like and oligodendrocyte-like states in LGG275 cells**

(A) Log2(FoldChange) of astrocyte and oligodendrocyte gene markers expression in CD44^+^GLAST^+^ (purple) and CD44^-^GLAST^-^ (orange) populations. Unsorted cells were used as control to calculate the Log2(FoldChange) (bars = mean, dots = n = 3) (* p<0,05, multiple Student test) (B) GSEA analysis of RNA sequencing comparing CD44^+^GLAST^+^ versus CD44^-^GLAST^-^ populations shows enrichment of astrocyte signatures in CD44^+^GLAST^+^ cells, and OPC and NPC metaprogram signatures in CD44^-^GLAST^-^ cells (n=3). NES = Normalized Enrichment Score, FDR = False Discovery Rate. q.value has been calculated using the Benjamini-Hochberg method.

**Figure S8 (related to Figure 5): Proteomic analysis of of astrocyte-like and oligodendrocyte-like states**

(A) 1D enrichment and fisher analyses in CD44^+^GLAST^+^ versus CD44^-^GLAST^-^ cells (n=3). Bars represent the median enrichment. All signaling pathways are significant a p.value <0,01 as determined by a Fisher’s exact test (B) Volcano plots showing proteins differentially expressed between CD44⁺GLAST⁺ and CD44⁻GLAST⁻ cells, focusing on markers associated with quiescent NSC1/NSC2 and active NSC phenotypes. Colored dots indicate significantly differentially expressed proteins (Student’s *t*-test with permutation-based FDR correction; FDR = 0.05, s₀ = 0.1), with purple indicating proteins enriched in CD44⁺GLAST⁺ cells and orange indicating proteins enriched in CD44⁻GLAST⁻ cells. Black dots represent non-significant proteins.

**Figure S9 (related to Figure 6): Astrocyte-like cells display plasticity and can transition toward an oligodendrocyte/NPC-like state in the presence of growth factors**

(A, B) IF analysis of APOE and OLIG2 expression in (A) FACS-sorted CD44^+^GLAST^+^ and (B) CD44^-^GLAST^-^ LGG275 cells grown with GF. Representative images were acquired at days 1, 6, 18, and 30 post-sorting, identifying astrocyte-like (APOE⁺OLIG2⁻; purple arrows) and oligodendrocyte/NPC-like (APOE⁻OLIG2⁺; orange arrows) cells. scale bar = 20µm. Right panels: quantification of each phenotype as a percentage of Hoechst⁺ nuclei; (dots on curve = mean ± S.E.M, n=3)

**Figure S10 (related to Figure 8): DLL3 expression and overexpression in LGG275 cells**

(A)  scVelo embedding of single-cell RNA-seq data from LGG275 cells cultured with growth factors (GF) or after GF withdrawal (no GF), showing DLL3 expression across annotated cell states (astrocyte-like, oligodendrocyte-like, NPC-like, and unknown). DLL3 expression is enriched in, and largely restricted to, the oligodendrocyte-like population (B) IF analysis of OLIG2 (green), DLL3 (magenta), and KI67 (yellow) in LGG275 cells cultured with growth factors (GF) or after GF withdrawal (no GF). Images illustrate DLL3 expression in both oligodendrocyte/NPC-like (OLIG2⁺) and OLIG2⁻ populations, as well as KI67 labelling among DLL3⁺ cells. Scale bar, 50 µm. Right panels: quantification of OLIG2/DLL3 and KI67/DLL3 subpopulations as % of Hoechst ^+^ cells (n=1, count = 500 cells) (C) IF analysis of P27 and DLL3 expression in LGG275 cells grown with or without GF, showing DLL3^+^ quiescent (P27^+^) or non-quiescent (P27^-^) cells, scale bar = 50µm. Right panel: Barplot shows quantification as % of Hoechst ^+^ cells (n=1, count = 500 cells) (D) Volcano plot of differentially expressed genes comparing DLL3-overexpressing cells versus control (CTL). significantly upregulated genes in DLL3 are highlighted in orange and genes upregulated in Ctrl in black (n=3) (E) Heatmap showing log2(Fold change) in the expression of selected proliferation-associated genes in DLL3-overexpressing cells relative to control (DLL3 vs Ctrl) (F) Heatmap showing log2(Fold change) in the expression of selected oligodendrocyte-lineage, stemness, and astrocyte markers in DLL3-overexpressing cells relative to control (DLL3 vs Ctrl).  All genes shown are significantly differentially expressed (P < 0.05), except those labeled “ns” (not significant) (G) Cytoscape visualization of enrichment signaling pathways between DLL3 overexpression vs Ctrl. P.value<0.05 and FDR q.value < 0.25 as determinate by the Benjamini-Hochberg method (n=3), with node size proportional to gene set size and node color intensity reflecting the magnitude of enrichment. Edges indicate gene overlap between gene sets (n = 3).

**Figure S11 (related to Figure 8): DLL3 overexpression leads a decrease of “astrocyte-like” GFAP^+^ in LGG336 cell lines**

(A) IF analysis of DLL3 (magenta) and KI67 (yellow) in the LGG336 cell line transduced with a GFP–DLL3 lentiviral vector and cultured with growth factors (GF). Representative images show DLL3⁺ cells (white arrows) and mitotic KI67⁺ cells (yellow arrows) in the DLL3-overexpression and Ctrl condition. Right panel: Barplot shows quantification as % of Hoechst^+^ cells (bars = mean, dots = n = 3) (*p<0.05, Dunnett’s multiple comparison test) (B) EDU incorporation analysis identifying proliferative EDU^+^ cells in DLL3 overexpressing cell vs Ctrl cells. bar plots show quantification as % of EDU⁺ cells (Bars = mean, dots = n = 3) (*p<0.05, Student’t test) (C) IF analysis of GFAP (magenta) in LGG336 cells transduced with a GFP–DLL3 lentiviral vector and cultured with growth factors (GF), comparing control (Ctrl) and DLL3-overexpressing conditions to quantify the astrocyte-like (GFAP⁺) population. Scale bar, 50 µm. Right panel: quantification of GFAPt ot cells as % of Hoechst^+^ cells (bars = mean, dots = n = 3) (*p<0.05, Dunnett’s multiple comparison).
